# What impact could DMPA use have had in South Africa and how might its continued use affect the future of the HIV epidemic?

**DOI:** 10.1002/jia2.25414

**Published:** 2019-11-15

**Authors:** Leo Beacroft, Jennifer A Smith, Timothy B Hallett

**Affiliations:** ^1^ MRC Centre for Global Infectious Disease Analysis Department of Infectious Disease Epidemiology Imperial College London London United Kingdom

**Keywords:** heterosexual transmission, HIV acquisition risk, mathematical models, contraceptive methods, women, South Africa

## Abstract

**Introduction:**

Some studies suggest that use of the injectable contraceptive depot medroxyprogesterone acetate (DMPA) may increase susceptibility to HIV infection. We aim to determine the influence that such an association could have had on the HIV epidemic in South Africa.

**Methods:**

We simulate the heterosexual adult HIV epidemic in South Africa using a compartmental model stratified by age, behavioural risk group, sex, male circumcision status and contraceptive use. We model two possible scenarios: (1) The “With Effect” scenario assumes that DMPA increases susceptibility to HIV infection by 1.20‐fold (95% confidence interval 1.06 to 1.36) based on a combination of the results of a recent randomised controlled trial (ECHO trial) and a number of observational studies. (2) The “No Effect” scenario assumes that DMPA has no effect on HIV acquisition risk. We calculate the difference in HIV‐related outcomes between the With Effect and No Effect scenarios to determine the potential impact that DMPA use could have had on the HIV epidemic.

**Results:**

A causal association between DMPA and HIV acquisition could have caused 430,000 (90% of model runs 160,000 to 960,000) excess HIV infections and 230,000 (90,000 to 470,000) AIDS deaths in South Africa from 1980 to 2017. These figures represent 4.3% (1.6% to 9.6%) and 6.9% (2.6% to 15.2%) of the total modelled estimates of HIV infections and AIDS deaths respectively in South Africa in that period. Of the additional infections, 36% (25% to 48%) would have occurred among men. If DMPA use continues at current levels, a potential causal association could cause an additional 130,000 (50,000 to 270,000) infections between 2018 and 2037. The excess infections would have required an additional 640,000 (190,000 to 1,660,000) years of ART from 1980 to 2017, and a further 2,870,000 (890,000 to 7,270,000) years of ART from 2018 to 2037.

**Conclusions:**

If there is a causal association between DMPA use and HIV risk, it could have substantially increased the scale of the HIV epidemic in South Africa, affecting not only the users of DMPA, but also their partners and the wider population. The magnitude of this potential effect demands careful data collection and a careful consideration of policy choices for contraception in settings with large HIV epidemics.

## 1 Introduction

Injectable hormonal contraception (IHC) is used by over 50 million women worldwide 1, the majority of whom are using depot medroxyprogesterone acetate (DMPA). It is particularly popular in southern and eastern Africa, for example in South Africa 17.7% of married and sexually active unmarried women use DMPA 2. However, some evidence suggests that there is a link between DMPA use and increased risk of acquiring HIV.

The available evidence consists of observational studies that have re‐analysed data that was initially collected for another purpose and so most studies contain some methodological limitations. A recent meta‐analysis including 14 studies with the fewest methodological limitations suggests that DMPA may increase the risk of HIV acquisition by a factor of 1.4 (95% confidence interval 1.23 to 1.59). Conversely, collective evidence suggests that oral contraceptive pills, injectable norethisterone enanthate (NET‐EN) and implants (for which limited data is available) do not affect HIV acquisition risk 3.

A number of animal and ex‐vivo studies have investigated possible biological mechanisms that would explain this association. There is evidence to suggest that the progestin medroxyprogesterone acetate (MPA) has been shown to inhibit parts of the immune system, and furthermore MPA may increase the frequency of target cells for HIV in the genital tract. These immunomodulatory effects could allow the virus to more easily infect target cells and avoid detection and removal by the immune system. MPA has also been shown to make the female reproductive tract more permeable, potentially allowing HIV to move through the lining of the reproductive tract more easily 4.

The World Health Organisation has updated its medical eligibility criteria for injectable contraceptives, including both DMPA and NET‐EN, in response to this evidence from a category 1 – which states that there should be “no restrictions on the use of the contraceptive method” – to a category 2 – which generally indicates that “the advantages of using the contraceptive method generally outweigh the theoretical or proven risks,” and advises that women at risk of HIV infection wishing to use injectable contraceptives should be counselled as to its potential side effects 5.

Based on the uncertainty surrounding the observational evidence, the Evidence for Contraceptive Options and HIV Outcomes (ECHO) trial, randomized 7829 HIV‐negative women to either DMPA, a copper intrauterine device (IUD) or a levonorgestrel (LNG) implant and followed them for up to 18 months throughout the trial, testing for HIV seroconversion every three months 6. The trial results have recently been released and showed no significant association between use of any of the contraceptive methods and HIV acquisition risk.

The results of the ECHO trial reduce the likelihood that DMPA use increases HIV risk, particularly for a “large” increase in risk of 1.5‐fold – the effect size that the trial was powered to detect. However, it does not conclusively disprove that a causal association exists. It is important to consider all available evidence, and so we have updated our understanding of the likelihood that a causal association exists and the effect size of such an association. An increase in risk by a factor of between 1 and 1.5 may still be a substantial increase in risk, particularly in areas of high HIV incidence.

To understand the impact that DMPA may have had on the HIV epidemic so far, and the potential future impact, it is important to consider the context of a complex epidemic including men and women of different ages and sexual behaviours. The impact would be greatest in those countries that have had historically high rates of DMPA use, as well as high HIV prevalence. DMPA has been available in South Africa since 1963 7, and has been widely used for more than 30 years 8. Furthermore, South Africa has the largest number of people living with HIV of any country worldwide, with adult prevalence of more than 15% since 2002 9. Therefore, a possible effect of DMPA use on HIV risk, even if the effect size is lower than the 1.5‐fold increase that the ECHO trial was powered to detect, could have particularly large implications in South Africa. To estimate the possible effects of such a causal association, we perform a mathematical modelling analysis comparing scenarios in which DMPA increases HIV susceptibility to scenarios in which there is no effect of DMPA use on HIV risk.

## 2 Methods

We use a deterministic compartmental mathematical model of heterosexual HIV transmission in South Africa 10, 11. The population is stratified by age, behavioural risk, sex, male circumcision status and contraceptive use. The population age structure is based on previous model projections of the South African population, based on single year age groups 12. The HIV‐infected population is divided into nine compartments based on stage of infection, CD4 count and treatment status. HIV‐infected individuals in the model are able to begin antiretroviral therapy (ART) at different stages of infection. The population is stratified into three behavioural risk groups, which have different rates of partner change, frequencies of sex acts and condom use. Rates of HIV transmission are dependent on the risk group of each partner, the treatment status of the HIV positive partner, the circumcision status of the HIV negative male partner, the infection stage of the HIV positive partner, and the contraceptive use of the female partner. For a full description of the HIV transmission within the model, see the Supporting Information.

We calibrate our model using data on age and sex‐specific HIV prevalence, total HIV incidence and incidence in high risk women (see Supporting Information). A number of parameters were fitted including those related to the per sex act probability of HIV transmission, rates of mixing between different behavioural risk groups, the size of these risk groups and the start time of the epidemic.

Injectable contraceptives have been widely used in South Africa for the majority of the HIV epidemic, the 1987 to 1988 DHS reported that 19.6% of married women were using injectable methods 8. Estimates of contraceptive use between different time points are often incompatible due to differences in methodologies between surveys; here we calibrate contraceptive use in the model to data from the 2016 Demographic and Health Survey (DHS) 2 and assume constant contraceptive total prevalence (including the prevalence of DMPA use) and age‐specific prevalence in South Africa over the period of observation (1980 to 2037). We believe that these assumptions are acceptable since injectables have been widely used throughout periods of high HIV prevalence in South Africa. We also believe that a substantially different pattern of age‐specific prevalence is unlikely.

We assume that scale‐up of male circumcision continues such that 60% of men aged 15 to 49 are circumcised by 2037 and ART scale‐up continues such that the 90‐90‐90 targets are reached by 2032. We ran the model 20,000 times and used a filtration method to select 100 acceptable epidemic fits. We then perform 1000 pairwise runs of the model, sampling from the set of fitted epidemic parameters each time. For each sampled parameter set, the model is run twice; in one run we assume that DMPA increases HIV susceptibility by a factor randomly sampled from a log‐normal hazard ratio (HR) distribution (“With Effect” scenario) and in the other we assume a hazard ratio of 1 and hence no effect of DMPA use on HIV risk (“No Effect” scenario). Due to the uncertainty surrounding the true association between DMPA and HIV risk, in the With Effect scenario we use a HR distribution with a median of 1.20 (2.5th to 97.5th percentiles 1.06 to 1.36) This distribution is based on a combination of the recent ECHO trial results, which report a HRs for DMPA use of 1.04 and 1.23 compared with the copper IUD and LNG implant respectively, and the most recent meta‐analysis of observational studies which found a mean HR of 1.40 for DMPA use. In using this distribution we acknowledge that the ECHO trial suggests that there is no substantial causal association between DMPA use and HIV risk. However, we also incorporate the body of observational evidence that suggests that such an association does exist. The ECHO trial was powered to detect a relative increase in risk of 1.5 13 and therefore the HR distribution represents a plausible range of values that may not have been detected by the ECHO trial.

For each parameter set we estimate two different epidemic curves and hence we are able to estimate the impact of DMPA use on the HIV epidemic in South Africa, under the assumption given by the current body of evidence. In this way, our credible range represents uncertainty due to the epidemic fit and the effect of DMPA use on the risk of HIV acquisition.

Full details of the parameter values used, model specification and calibration can be found in the Supporting Information.

## 3 Results

**Figure 1:**
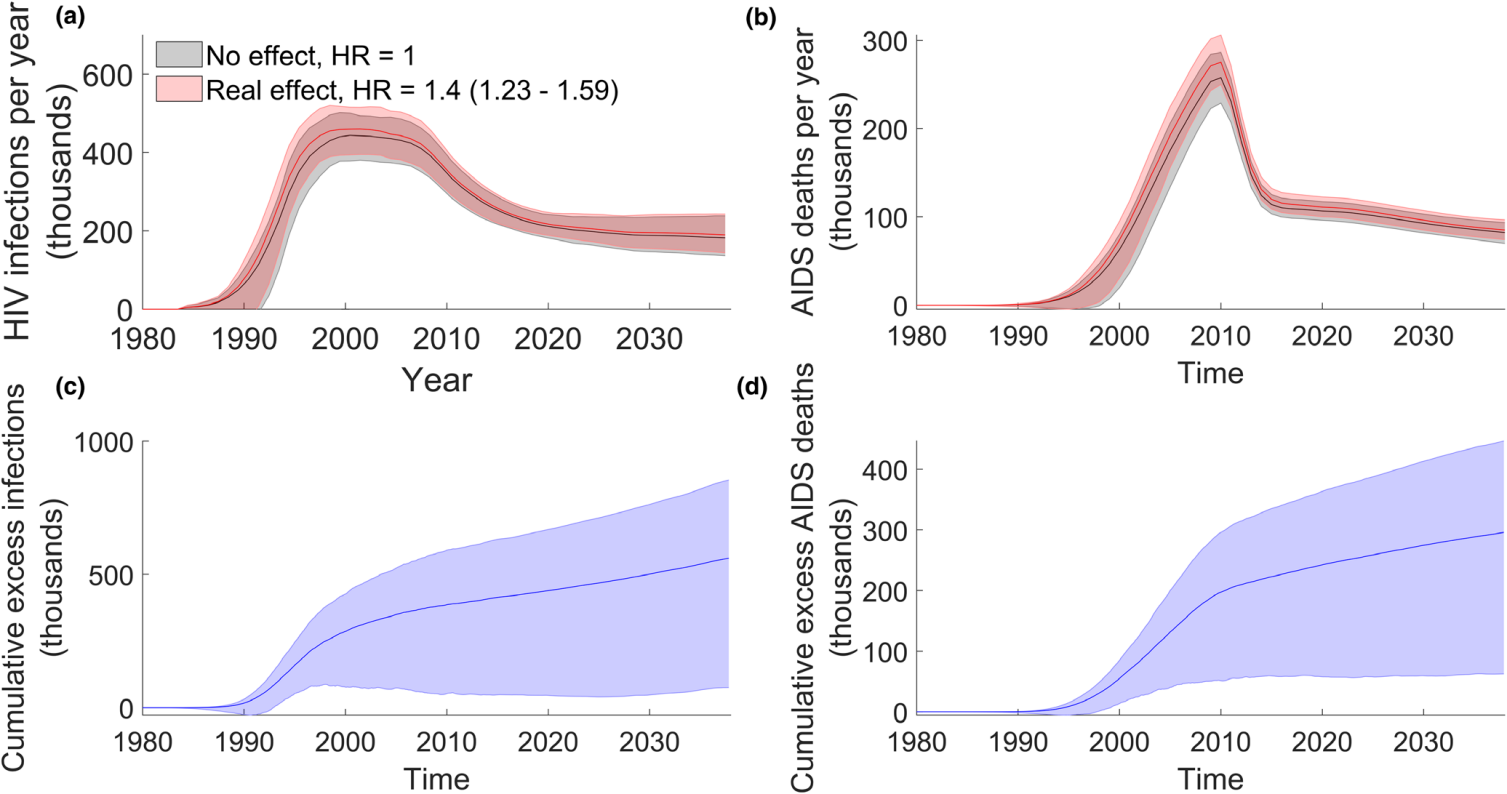
Excess infections and AIDS deaths. In each panel the line represents the median of model results, and the shaded area represents the 5 to 95th percentiles of model runs. (Top left(a)) HIV infections per year in South Africa. Results in red assume a causal association between DMPA use and HIV susceptibility (With Effect scenario), results in grey assume no effects from DMPA use (No Effect scenario). Shaded area represents the 90% of the model variation. (Top right(b)) AIDS deaths per year for both the With Effect and No Effect scenarios. (Bottom left(c)) Excess infections represent the median pairwise difference in infections between the With Effect and No Effect scenarios. The median cumulative excess infections are shown by the blue line, with the shaded area representing the 90% of the model variation. (Bottom right(d)) Median and 90% of model variation for cumulative excess AIDS deaths per year.

Figure 1a shows that for simulations in which DMPA increases HIV susceptibility, the epidemic is larger relative to the No Effect scenario. We observe a median excess 430,000 (90% of model runs 160,000 to 960,000) infections from 1980 to 2017, which represents 4.3% (1.6% to 9.6%) of the total HIV infections in this period (Table 1). Of these, we predict that on average, 275,000 occur in women and 155,000 occur in men. The fraction of excess infections that occur in men is 36% (90% of model runs 25% to 48%). The extra infections caused by the potential increase in HIV susceptibility would necessitate a median estimate of 640,000 (190,000 to 1,660,000) extra years of ART from 1980 to 2017.

**Table 1:** Key statistics

	1980 to 2017 (90% of model runs)	2018 to 2037 (90% of model runs)
Excess infections	430,000 (160,000 to 960,000)	130,000 (50,000 to 270,000)
Fraction of excess infections in men	36% (25% to 48%)	36% (24% to 47%)
Excess AIDS deaths	230,000 (90,000 to 470,000)	60,000 (20,000 to 150,000)
Additional years of ART	640,000 (190,000 to 1,660,000)	2,870,000 (890,000 to 7,270,000)
Population attributable fraction (PAF) of infections due to DMPA use	4.3% (1.6% to 9.6%)	3.3% (1.2% to 7.3%)
PAF of AIDS deaths due to DMPA use	6.9% (2.6% to 15.2%)	5.6% (2.1% to 12.4%)
PAF of years of ART due to DMPA use	3.3% (1.0% to 8.5%)	2.9% (0.9% to 7.4%)

Output statistics are presented in terms of medians and associated 90% model variation (5th to 95th percentiles of model runs). Excess infections, deaths and additional years of ART represent the difference in the respective outputs between the With Effect and No Effect scenarios. The population attributable fraction (PAF) is the fraction of infections, AIDS deaths, or years of ART in the With Effect scenario that are attributable to the effects of DMPA use on HIV susceptibility. For each pair of model runs, the PAF is the difference in the number of infections, AIDS deaths, or years of ART between the With Effect and the No Effect scenarios, calculated as a percentage of the total infections, AIDS deaths, or years of ART respectively in the With Effect scenario.

Furthermore, we see an increase in AIDS deaths in the With Effect scenario (Figure 1) and estimate that a causal association between DMPA and HIV risk would have caused a median 230,000 additional deaths from AIDS (90,000 to 470,000) to date. This represents 6.9% (2.6% to 15.2%) of the AIDS deaths in this period.

Projecting forward, from 2018 to 2037 we predict an excess 130,000 (90% of model runs 50,000 to 270,000) infections between 2018 and 2037 as well as 60,000 (20,000 to 150,000) additional deaths. As a result of the excess HIV infections an extra 2,870,000 (890,000 to 7,270,000) years of ART would be required from 2018 to 2037 (Table 1).

## 4 Discussion

The use of DMPA may have had a profound impact in exacerbating the spread of HIV in South Africa. Its use could be responsible for 4.3% of infections to date. The impact would most heavily affect the female users of DMPA themselves, but additional infections would also occur in male partners of DMPA users and in the wider population.

The major limitation of this analysis is the uncertainty on the true association between DMPA use and HIV susceptibility. We have therefore sampled HRs from a distribution, to incorporate the significant body of observational evidence, suggesting a mean HR of 1.4, and the ECHO trial, which produced HRs of 1.04 for DMPA compared to the copper IUD and 1.23 for DMPA compared with the LNG implant. Whilst the ECHO trial provides the best evidence of the available studies, the trial was powered to detect a hazard ratio of 1.5, and so it is still possible that DMPA does increase HIV risk, but this was not detected by the trial. This becomes more likely for HRs, such as 1.2 (the median of the HR used in this modelling analysis) which would still represent a substantial increase in risk in high‐incidence areas. We therefore believe that the HR distribution used in this study represents credible uncertainty surrounding the possible causal association of DMPA use on HIV acquisition risk.

There is also some uncertainty regarding scale up of contraception. DMPA was introduced in South Africa in the 1960s 7 and, in a 1977 to 1978 national survey injectable contraceptives (including DMPA) were reported to be used by 19.6% of women married or in a union 8. Due to incompatibility between different contraceptive prevalence estimates, we assume constant contraceptive use throughout the HIV epidemic. This is a limitation of our analysis, but since DMPA has been widely used throughout the HIV epidemic in South Africa, changes in contraceptive use are unlikely to be large, and so we would not expect a large impact on the results of our analysis.

Our model assumes 17.7% of women use DMPA, based on estimates of contraceptive prevalence from 2016 2. If DMPA use has increased during the course of the HIV epidemic, and there continues to be increased use in the future, then our results will overestimate the past impact of a possible effect of DMPA use on HIV risk, but will underestimate future impact. The contraceptive age‐profile of the model is based on national survey estimates, DMPA use is highest for women in their 20s and 30s and lower for women under 20 and over 40 2. If DMPA use was in fact most commonly used by women in their 40s, for whom HIV incidence is lower, then the impact of the putative effect of DMPA on the HIV epidemic would be lower. However, we believe it is unlikely that such a substantially different age‐profile of DMPA use could occur.

While we have focussed on South Africa, this research has implications for many other countries. The impact of a potential causal association would vary between countries depending on a number of factors including, the pattern of DMPA use in the country, the size and timing of the HIV epidemic and the scale up of interventions. For example, in Zambia, there is a large HIV epidemic, with 11.5% of the adult population living with HIV in 2017 9. DMPA use is also high, with the most recent Demographic and Health Survey reporting that 17.9% of married women used the method in 2013 to 2014 14. However, DMPA use has largely been expanded in the 21st century, particularly since 2007. Therefore there would be a shorter period of overlap between DMPA use and the HIV epidemic in Zambia; the number of new HIV infections peaked in the mid‐1990s and AIDS deaths had peaked by 2003 9, at which point between 4.5% to 8.5% of married women were using DMPA 14. Since much of the scale up of DMPA in Zambia happened after the peak of the HIV epidemic, we would expect a potential causal association between DMPA use and HIV risk to contribute a smaller fraction of HIV‐related outcomes in Zambia, when compared with South Africa, where DMPA had been used at high levels since the late 1980s. We would predict that a causal association between DMPA use and HIV risk, if it exists, to be responsible for a larger fraction of HIV‐related outcomes in countries where DMPA has been used extensively throughout the HIV epidemic. In countries across southern and eastern Africa, DMPA accounts for a large fraction of the contraceptive method mix and many of these countries have also had high HIV prevalence. It is likely that any effects of DMPA on HIV risk would be setting specific and depend on many factors, including the temporal overlap of HIV incidence and patterns of DMPA in particular age groups.

The use of a highly stratified mathematical model has a number of advantages. We account for age‐specific differences in HIV prevalence as well as contraceptive differences by age. One limitation is that the model does not capture the formation and dissolution of partnerships and so may introduce bias by not accounting for specific sexual behaviour patterns in individuals.

## 5 Conclusions

Discussion as to the relative benefits, and potential risks, of DMPA use are ongoing. The World Health Organization has made several updates to its guidelines for injectable contraceptive use for those at high risk of HIV 5, 15. We aim to aid discussion by providing estimates of the impact that DMPA use may have had on the HIV epidemic as well as projections for future impact if use is continued and a real risk exists. Our results suggest that such an association between DMPA use and HIV risk could have increased the number of HIV infections by 430,000 (90% of model runs 160,000 to 960,000) which could have caused an additional 230,000 (90,000 to 470,000) AIDS deaths in South Africa between 1980 and 2017. We estimate that on average, 36% (25% to 48%) of infections occurred in men, showing that the impact of an association between DMPA use and HIV risk would affect not only women using DMPA, but also their partners and the wider population.

In South Africa, many women have limited access to safe healthcare facilities, and hence the maternal mortality ratio is approximately 8 to 10 times higher than those of high income countries 16, 17. Hence a reduction in DMPA use, without transition to another equally effective method of contraception, could lead to an increase in unintended pregnancies, causing a range of negative consequences including maternal morbidity and mortality. DMPA remains a valuable contraceptive for women throughout the world, allowing women to control their fertility and avoid potential negative consequences, both health and socioeconomic, of unintended pregnancy. These results reiterate the need for governments and family planning service providers to establish broad contraceptive method mix, to allow women to safely manage both their HIV prevention and family planning needs.

## Competing interests

Mr Beacroft reports grants from USAID during the conduct of the study. Personal fees from Bill and Melinda Gates Foundation grants from Bill and Melinda Gates Foundation, outside the submitted work; Travel expenses from WHO outside submitted work. Dr Smith reports grants from USAID during the conduct of the study, personal fees from Bill and Melinda Gates Foundation grants from Bill and Melinda Gates Foundation, outside the submitted work. Dr Hallett reports grants USAID during the conduct of the study; grants from BMGF, World Bank, UNAIDS, Rush Foundation, Wellcome Trust, personal fees from BMGF, New York University, WHO, GFATM, outside the submitted work.

## Authors’ contributions

TH, JS and LB designed the analysis. LB performed the analysis. LB, JS and TB analysed the results. LB, JS and TB wrote the manuscript.

## Funding

The work reported in this article was supported by a grant from the United States Agency for International Development (USAID) through an award subcontracted through Abt Associates. We acknowledge joint Centre funding from the UK Medical Research Council and Department for International Development. Funding was also received from Bill & Melinda Gates Foundation.

## Supporting information


**Figure S1.** Natural history of HIV infection and ART initiation as represented in the model.
**Figure S2.** The proportion of adult men that are circumcised with respect to time. The level of circumcision in the model was calibrated to data reported in a nationally representative survey [11]
**Figure S3.** The percentage of HIV positive adults (15 to 49) receiving antiretroviral therapy in South Africa. Model data is compared to estimates of the percentage of HIV positive adults on ART in South Africa [18,19].
**Figure S4.** Total contraceptive prevalence calibration. Total contraceptive prevalence among 15 to 49 year old women is calibrated to nationally representative survey data [21]. The percentage of HIV positive adults (15 to 49) receiving antiretroviral therapy in South Africa. Model data is compared to estimates of the percentage of HIV positive adults on ART in South Africa [18,19].
**Figure S5.** Age‐specific contraceptive prevalence calibration. Contraceptive prevalence among different age groups are calibrated to nationally representative survey data [21].
**Figure S6.** Population pyramids for South Africa for 1985, 1990, 1995, 2000, 2005 and 2010. Model population structure is compared to annual age‐structured population size model estimates produced by the Actuarial Society of South Africa [10].
**Figure S7.** Population size with respect to time. The total population of the model was calibrated to previous estimates from a demographic model of the South African population [10].
**Figure S8.** HIV incidence in 15 to 49 year olds. HIV incidence in adults was calibrated to incidence data from a nationally representative survey as well as incidence estimates produced by a mathematical model calibrated to prevalence data [11]. Blue dotted lines represent 10th , 50th and 90th percentiles of model variation.
**Figure S9.** Prevalence in 15 to 49 year olds. Adult HIV prevalence is calibrated to nationally representative survey data from South Africa as well as UNAIDS prevalence estimates [11,19,22]. Blue dotted lines represent 10th , 50th and 90th percentiles of model variation.
**Figure S10.** Male HIV prevalence. HIV prevalence in the model was calibrated to sex‐specific prevalence data [11]. Blue dotted lines represent 10th , 50th and 90th percentiles of model variation.
**Figure S11.** Female HIV prevalence. HIV prevalence in the model was calibrated to sex‐specific prevalence estimates [11] . Blue dotted lines represent 10th , 50th and 90th percentiles of model variation.
**Figure S12.** Male HIV incidence. HIV incidence in the model was calibrated to sex‐specific incidence estimates produced by a mathematical model calibrated to nationally representative prevalence data [11]. Blue dotted lines represent 10th , 50th and 90th percentiles of model variation.
**Figure S13.** Female HIV incidence. HIV incidence in the model was calibrated to sex‐specific incidence estimates produced by a mathematical model calibrated to nationally representative prevalence data [11]. Blue dotted lines represent 10th , 50th and 90th percentiles of model variation.
**Table S1.** Natural history of infection parameters
**Table S2.** Behavioural parameters and values
**Table S3.** Factor increments in transmission probability per sex act with respect to baseline transmission probability (β_0_)
**Table S4.** Contraceptive efficacy and continuation rates for methods used in the model.
