# Supplementary material for: What impact could DMPA use have had in South Africa and how might its continued use affect the future of the HIV epidemic?
Source: J Int AIDS Soc. 2019 Nov 15;22(11):e25414. doi: 10.1002/jia2.25414 (PMC6856612; doi:10.1002/jia2.25414)
Supplement: Supplementary file 1 — Figure S1. Natural history of HIV infection and ART initiation as represented in the model. Figure S2. The proportion of adult men that are circumcised with respect to time. The level of circumcision in the model was calibrated to data reported in a nationally representative survey [11] Figure S3. The percentage of HIV positive adults (15 to 49) receiving antiretroviral therapy in South Africa. Model data is compared to estimates of the percentage of HIV positive adults on ART in South Africa [18,19]. Figure S4. Total contraceptive prevalence calibration. Total contraceptive prevalence among 15 to 49 year old women is calibrated to nationally representative survey data [21]. The percentage of HIV positive adults (15 to 49) receiving antiretroviral therapy in South Africa. Model data is compared to estimates of the percentage of HIV positive adults on ART in South Africa [18,19]. Figure S5. Age‐specific contraceptive prevalence calibration. Contraceptive prevalence among different age groups are calibrated to nationally representative survey data [21]. Figure S6. Population pyramids for South Africa for 1985, 1990, 1995, 2000, 2005 and 2010. Model population structure is compared to annual age‐structured population size model estimates produced by the Actuarial Society of South Africa [10]. Figure S7. Population size with respect to time. The total population of the model was calibrated to previous estimates from a demographic model of the South African population [10]. Figure S8. HIV incidence in 15 to 49 year olds. HIV incidence in adults was calibrated to incidence data from a nationally representative survey as well as incidence estimates produced by a mathematical model calibrated to prevalence data [11]. Blue dotted lines represent 10th , 50th and 90th percentiles of model variation. Figure S9. Prevalence in 15 to 49 year olds. Adult HIV prevalence is calibrated to nationally representative survey data from South Africa as well as UNAIDS prevalence estimates [1 [file JIA2-22-e25414-s001.docx]

What impact could DMPA use have had in South Africa and how might its continued use affect the future of the HIV epidemic?

**Supporting Information**

# Model overview

Based on previous model developed by Cremin and co-authors [1,2], we adapted a deterministic compartmental model defined by a set of ordinary differential equations. It is designed to represent heterosexual HIV transmission at the population level in South Africa, a mature, generalised HIV epidemic. Our aim is to estimate how a possible interaction between use of the injectable contraceptive depot medroxyprogesterone acetate and susceptibility to HIV infection could have impacted the HIV epidemic in South Africa, and how such an association could continue to impact the HIV epidemic in the future.

The model population is divided into compartments that are distinguished by sex, circumcision status (if male), age, infection stage, sexual behaviour and contraceptive use, with events (e.g. HIV infection, death, ART initiation etc.) represented as movement between these compartments [3–5]. Heterogeneity in sexual behaviour is incorporated in the model by stratifying men and women into three risk groups according to their average effective partnership formation rate.

A full description of the model structure, parameter values used and calibration is provided under the following sections: 2.2 Natural History of HIV infection, 2.3 Demography, 2.4 HIV transmission and sexual mixing, 2.5 Male circumcision, 2.6 Antiretroviral treatment, 2.7 Contraception, 2.8 Reproductive Health Outcomes, 2.9 Model calibration.

Our model assumes that the sexually active population is aged 15-49 years. Many of the equations describing the HIV transmission process use subscript “a”, “ a’ ”, “A” or “ A’ ” to denote an individual’s age, their partner’s age, the individual’s five year age-group or their partner’s five year age-group respectively. Since all processes concerning sexual transmission of HIV pertain only to the 15-49 year old population we have chosen not to replace the age subscript with “15-49” in order to aid clarity by reducing equation length. Similarly, for ease of notation, we have not included subscript “t” to denote time, which remains to all state variables and dependent functions.

# Natural history of HIV infection

A flow diagram for the natural history of HIV infection and treatment cascade is shown in Figure S1.


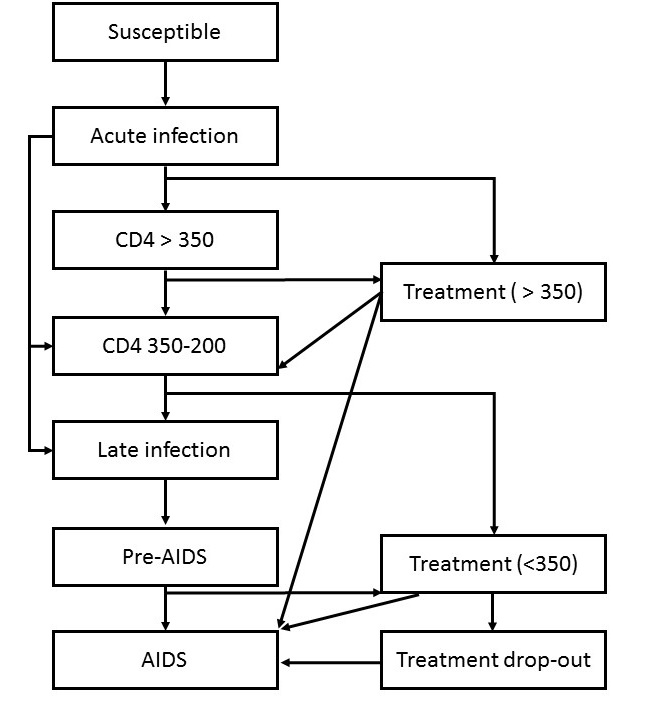


*Figure S1: Natural history of HIV infection and ART initiation as represented in the model*

*Movement between compartments is indicated by arrows.*

The model is specified by the following ordinary differential equations:

The equations describing susceptible individuals are:

$\frac{dX_{l,k,1}^{0,p}}{dt}=b_{a}N\Psi_{\left( l,k,p,1 \right)}-\left( \lambda_{l,k,1}^{p}+\mu_{k,1} \right)X_{l,k,1}^{0,p}+f\left( 1 \right)+g(k)+ q(p)$ for a=1

$\frac{dX_{l,k,a}^{0,p}}{dt}=-\left( \lambda_{l,k,A}^{p}+\mu_{k,a} \right)X_{l,k,a}^{0,p}+f\left( a \right)+g(k)+ q(p)$ for a≠1

(1)

The equations describing HIV infected individuals are:

$$\frac{dX_{l,k,a}^{1,p}}{dt}=\lambda_{l,k,A}^{p}X_{l,k,a}^{0,p}-\left( {(\gamma1+\gamma2+\gamma3)\sigma}_{1}+\mu_{k,a} \right)X_{l,k,a}^{1,p}+f\left( a \right) + g(k)+ q(p)$$

$$\frac{dX_{l,k,a}^{2,p}}{dt}=\left( 1-\phi_{1} \right)\sigma_{1}{\gamma1X}_{l,k,a}^{1,p}-\left( \sigma_{2}+\mu_{k,a} \right)X_{l,k,a}^{2,p}+f\left( a \right) + g(k)+ q(p)$$

$$\frac{dX_{l,k,a}^{3,p}}{dt}=\left( 1-\phi_{2} \right)\sigma_{2}\gamma2X_{l,k,a}^{2,p}+\zeta_{E}X_{l,k,a}^{7,p}-\left( \sigma_{3}+\mu_{k,a} \right)X_{l,k,a}^{3,p}+f\left( a \right)+ g(k)+ q(p)$$

$$\frac{dX_{l,k,a}^{4,p}}{dt}=\left( 1-\phi_{3} \right)\sigma_{3}\gamma3X_{l,k,a}^{3,p}-\left( \sigma_{4}+\mu_{k,a} \right)X_{l,k,a}^{4,p}+f\left( a \right)+ g(k)+ q(p)$$

$$\frac{dX_{l,k,a}^{5,p}}{dt}=\sigma_{4}X_{l,k,a}^{4,p}-\left( \sigma_{5}+\mu_{k,a} \right)X_{l,k,a}^{5,p}+f\left( a \right)+ g(k)+ q(p)$$

$$\frac{dX_{l,k,a}^{6,p}}{dt}=\left( 1-\phi_{4} \right)\sigma_{5}X_{l,k,a}^{5,p}+\omega_{E}X_{l,k,a}^{7,p}+\omega_{L}X_{l,k,a}^{8,p}+\tau_{D}X_{l,k,a}^{9,p}-\left( \Omega+\mu_{k,a} \right)X_{l,k,a}^{6,p}+f\left( a \right)\ldots+ g\left( k \right)+ q(p)$$

$$\frac{dX_{l,k,a}^{7,p}}{dt}= {\phi_{1}\sigma}_{1}\gamma1X_{l,k,a}^{1,p}+{\phi_{2}\sigma}_{2}X_{l,k,a}^{2,p}-\left( \omega_{E}+\zeta_{E}+\mu_{k,a} \right)X_{l,k,a}^{7,p}+f\left( a \right)+ g(k)+ q(p)$$

$$\frac{dX_{l,k,a}^{8,p}}{dt}={\phi_{3}\sigma}_{3}X_{l,k,a}^{3,p}+{\phi_{4}\sigma}_{5}X_{l,k,a}^{5,p}-\left( \omega_{L}+\zeta_{L}+\mu_{k,a} \right)X_{l,k,a}^{8,p}+f\left( a \right)+ g(k)+ q(p)$$

$$\frac{dX_{l,k,a}^{9,p}}{dt}=\zeta_{L}X_{l,k,a}^{8,p}-\left( \tau_{D}+\mu_{k,a} \right)X_{l,k,a}^{9,p}+f\left( a \right)+ g(k)+ q(p)$$

(2) For Natural History of Infection Parameter definitions and values see Table S1 (pages 8-9).

$X_{l,k,a}^{s,p}$ represents the number of individuals of stage of infection *s*, contraceptive use *p*, risk group *l*, sex / male circumcision status *k*, and age *a*. f(a) represents aging in the model (page 10). q(p) denotes movement of women between contraceptive types (page 28). g(k) denotes movement of men from the uncircumcised to the circumcised compartments of the model (page 25). λ represents the force of infection (page 12).

Stages of infection denoted by s, are: 0 - susceptible. 1 acute HIV infection, 2 – HIV infected CD4 count > 350, 3 - HIV infected CD4 200-350, 4 - late stage HIV infection with CD4 < 200, 5 - pre-AIDS, 6 - AIDS, 7 – early-initiated ART (CD4 >350), 8 – late initiated ART (CD4 < 350), 9 – treatment drop-out.

For a given stage of HIV infection, hazards of progression to the next stage are given by the rates σ _(1,2,3,4,5)_. The late infection stage is defined by the mean time between when CD4 count falls below 200 cells/μl and viremic rebound 19 months, on average, before death. The pre-AIDS stage characterizes the 9 month period of heightened infectiousness before AIDS, which represents a 10 month period of no transmission risk [6].

Mortality in the AIDS stage is denoted by the parameter Ω. An important limitation of this model is that AIDS-related mortality only applies to this final AIDS stage. However, in reality some infected individuals may die of AIDS-related illnesses at higher CD4 counts.

Several representations of ART initiation are possible in the model; ART can be initiated following acute infection, when an individual’s CD4 count drops below 350, 200, or 100 cells per microliter. The proportion of individuals initiating ART following acute infection, at CD4 <350 cells/μl, CD4 <200 cells/μl and CD4 <100 cells/μl are controlled by the parameters *ϕ_1,_ ϕ_2_*, *ϕ_3_* and *ϕ_4,_* respectively*_._*  ART initiation at low CD4 counts (< 100 cells/μl) is used to represent the initial pattern of ART initiation (i.e. for urgent clinical need), when ART was first introduced in South Africa.

ART is assumed to extend the survival of treated individuals (the increase in life expectancy depends on whether ART is initiated ‘≥350 cells/μl’ or ‘<350 cells/μl’) while reducing infectiousness [7,8] . Individuals initiating ART ≥350 cells/μl or <350 cells/μl are assumed to survive on average $\frac{1}{\omega_{E}}$ or $\frac{1}{\omega_{L}}$ years before progressing to AIDS, respectively. Drop outs from treatment initiated ≥350 cells/μl return to having a CD4 ≥350 cells/μl and progress through infection. Drop outs from treatment when initiated <350 cells/μl’ progress to AIDS after a period of slightly heightened infectiousness represented by the ‘Treatment drop-out’ compartment in Figure S1.

| Parameter | Symbol | Value | Source |
| --- | --- | --- | --- |
| Mean duration of acute infection | 1/σ_1_ | 0.25 years | [6] |
| Mean duration from the end of acute infection to CD4 350 cells/μl | 1/σ_2_ | 9.25 years |  |
| Mean duration from CD4 350 cells/μl to CD4 200 cells/μl | 1/σ_3_ | 3.54 years |  |
| Mean duration from CD4<200 cells/μl to viremic rebound | 1/σ_4_ | 1.12 years |  |
| Mean duration of viremic rebound before AIDS | 1/σ_5_ | 0.75 years |  |
| AIDS mortality rate | Ω | 1/0.833 (10 month period before death) |  |
| Proportion of HIV infected individuals starting infection at CD4> 350 cells/μl | $\gamma1$ | 0.81  (0.58+0.23) | [9] |
| Proportion of HIV infected individuals starting infection at CD4 200-350 cells/μl | $\gamma2$ | 0.16 |  |
| Proportion of HIV infected individuals starting infection at CD4<200 cells/μl | $\gamma3$ | 0.03 |  |
| Progression to AIDS from early treatment | $\omega_{E}$ | 0.0026 | Calibrated |
| Progression to from late treatment | $\omega_{L}$ | 0.0843 | Calibrated |
| Rate of progression to AIDS from treatment drop-out | $\tau_{D}$ | 0.2 year^-1^ | Calibrated |
| Drop-out rate from early treatment | $\zeta_{L}$ | 0.005 year^-1^ | Calibrated |
| Drop-out rate from late treatment | $\zeta_{E}$ | 0.005 year ^-1^ | Calibrated |
| Age-specific fertility rate | b_a_ | Variable | [10] |
| Total population | N | Variable |  |
| Demography of births | $\Psi_{\left( l,k,p,1 \right)}$ | See page 12 |  |
| Age- and sex-specific mortality | $\mu_{k,a}$ |  | [10] |

***Table S1: Natural history of infection parameters***

# Demography

The model is stratified by one-tenth of a single year of age from birth to 100 years. Ageing of individuals is represented by:

$$X_{l,k,1}^{0,p}= X_{l,1,a}^{s,p}b_{a} for a=1$$

$$X_{l,k,a}^{s,p}=X_{l,k,a-1}^{s,p} for a>1$$

(3)

$\Psi_{\left( l,k,p,a \right)}$ is the matrix of population distribution in the year the epidemic starts (*t_0_*) over each l, k, a stratum and it is defined in terms of: (i) $\varphi_{f(l)}$ and $\varphi_{m(l)}$which are the proportion of females and males respectively in each risk activity group; and (ii) *f_a_* which is the proportion of the population in each tenth of a year of age, with $\sum_{a=1}^{1000} f_{a}=1$. The parameter *f_cm_* gives the fraction of males who are circumcised.

The total number in the population (N) and $\Psi_{\left( l,k,p,a \right)}$ are given by:

$$N=\sum_{l=1}^{3} \sum_{k=1}^{3} \sum_{p=1}^{7} \sum_{a=1}^{1000} \left( X_{l,k,a}^{0,p}+X_{l,k,a}^{1,p}+X_{l,k,a}^{2,p}+X_{l,k,a}^{3,p}+X_{l,k,a}^{4,p}+X_{l,k,a}^{5,p}+X_{l,k,a}^{6,p}+X_{l,k,a}^{7,p}+X_{l,k,a}^{8,p}+X_{l,k,a}^{9,p} \right)$$

(4)

$$\Psi_{\left( l,k,p,a \right)}=\left\{ \begin{aligned} \frac{1}{2}\varphi_{f(l)}f_{p} f_{a} any l; k=1;any p; any a; \\ \frac{1}{2}\varphi_{m(l)}f_{a}\left( 1-f_{cm} \right) any l; k=2; p=1; any a; \\ \frac{1}{2}\varphi_{m(l)}f_{a}f_{cm} any l; k=3;p=1; any a; \end{aligned} \right.$$

(5)

Individuals enter the population as susceptible at birth (i.e. age zero), the distribution of whom is defined by the population distribution matrix over each *l, k and p* stratum ($\Psi_{\left( l,k,p,1 \right)}$), given by:

$$\Psi_{\left( l,k,1,1 \right)}=\left\{ \begin{aligned} \frac{1}{2}\varphi_{f\left( l \right)} any l; k=1;p=1 (no method); a=1 (0 years); \\ \frac{1}{2}\varphi_{m\left( l \right)}\left( 1-f_{cm} \right) any l; k=2;p=1 (no method); a=1 (0 years); \\ \frac{1}{2}\varphi_{m\left( l \right)}f_{cm} any l; k=3; p=1 (no method); a=1 (0 years); \end{aligned} \right.$$

(6)

Age-specific Age-specific fertility rates and age- and sex-specific non-AIDS mortality rates (*μ_k,a_*) are taken from the ASSA 2008 model and are updated each year from 1985 to 2025 as estimated by that model [10]. AIDS-related mortality is modeled explicitly (Table S1). The South African population distribution by age in 1985 (*f _a_*) is taken from the same source [10]. The fraction of the female population using each contraceptive method ($f_{p})$ is based on the South African National HIV Prevalence, Incidence and Behaviour Survey, 2012 [11].

# HIV transmission and sexual mixing

##### Force of infection

The force of infection is the per capita rate at which susceptible individuals acquire infection. Following previous work [1], the per capita force of infection $\lambda_{l,k,A}^{p}$ is the force of infection experienced by individuals of each contraceptive group, risk group, sex, circumcision status (if male) and five year age group from the infected population of the opposite sex at a given time. Characteristics of an individual (p, l, k and A (where A is five year age group)) are distinguished from those of their sexual partners by means of a prime (i.e. p’, l’, k’ and A’). The force of infection is calculated by five year age group and then applied to each single year of age in that group.

The force of infection depends on the pattern of partnership formation between different risk and five year age groups and on the probability of transmission per partnership as well as DMPA usage by female partners, and is defined as:

For women not using DMPA:

$$\lambda_{l,1,A}^{p}=\sum_{l'} \sum_{k'} \sum_{p'} \sum_{A'} \sum_{s'} \left[ C_{g,A,l}\rho_{g,A,l,A^{'},l^{'}}\left( \frac{X_{l^{'},k^{'},A^{'}}^{s^{'}, p^{'}}}{\sum_{k^{'}=2}^{3} \sum_{p'} \sum_{s'} X_{l^{'},k^{'},A^{'}}^{s^{'},p^{'}}} \right)Z_{l,1,p,s^{'},l^{'},k^{'},p^{'}} \right]$$

For women using DMPA:

$$\lambda_{l,1,A}^{3}=H\sum_{l'} \sum_{k'} \sum_{p'} \sum_{A'} \sum_{s'} \left[ C_{g,A,l}\rho_{g,A,l,A^{'},l^{'}}\left( \frac{X_{l^{'},k^{'},A^{'}}^{s^{'}, p^{'}}}{\sum_{k^{'}=2}^{3} \sum_{p'} \sum_{s'} X_{l^{'},k^{'},A^{'}}^{s^{'},p^{'}}} \right)Z_{l,1,3,s^{'},l^{'},k^{'},p^{'}} \right]$$

Where *H* is the hazard ratio of HIV acquisition risk for women using DMPA.

For men:

$$\lambda_{l,k,A}^{p}=\sum_{l'} \sum_{p'} \sum_{A'} \sum_{s'} \left[ C_{g,A,l}\rho_{g,A,l,A^{'},l^{'}}\left( \frac{X_{l^{'},1,A'}^{s^{'}, p^{'}}}{\sum_{p'} \sum_{s'} X_{l^{'},1,A^{'}}^{s^{'},p^{'}}} \right)Z_{l,k,p,s^{'},l^{'},1,p^{'}} \right]$$

(7)

Where:

$C_{g,A,l}$ defines the mean number of partnerships (see page 17)

$\rho_{g,A,l,A^{'},l^{'}}$ defines sexual mixing in partnerships (see page 16)

$Z_{l,k,p,s^{'},l^{'},1,p^{'}}$ defines the probability of transmission per partnership (see page 13)

##### Probability of transmission per partnership

The probability of transmission per partnership $Z_{l,k,p,s^{'},l^{'},k^{'},p^{'}}$ is defined as:

For women and for uncircumcised men:

$$Z_{l,k,1,s^{'},l^{'},1,p^{'}}=1-\left( \left( 1-\beta_{1,k}^{s^{'}k^{'}}{\varpi)}^{\Pi} \right)\left( (1-\beta_{1,k}^{s^{'}k^{'}})^{\bar{\Pi}} \right. \right)$$

For circumcised men:

$$Z_{l,3,1,s^{'},l^{'},k^{'},p^{'}}=1-\left( \left( 1-\beta_{1,3}^{s^{'}k^{'}}{\varpi)}^{\Pi} \right)\left( (1-\beta_{1,3}^{s^{'}k^{'}})^{\bar{\Pi}} \right. \right)$$

(8)

Where:

$$\Pi=U(l,l^{'})\bar{q}_{(t)}n_{sex}\left( l,l^{'} \right)$$

$$\bar{\Pi}=\left( 1-U(l,l^{'})\bar{q}_{\left( t \right)} \right)n_{sex}\left( l,l^{'} \right)$$

(9)

*Π* is the number of sex acts protected by condoms in a partnership between an individual of risk group *l* and their partner of risk group *l’* and $\bar{\Pi}$ is the number of sex acts not protected by condoms in a partnership between an individual of risk group *l* and their partner of risk group *l’*.

*n_sex_(l,l’)* is a matrix defining the number of sex acts in a partnership between an individual of risk group l and a partner of risk group l’.

*U(l,l’)* is a matrix defining the proportion of sex acts in which condoms are used in a partnership between an individual’s risk group l and their partner’s risk group l’, modulated by any increase in condom use due to changes over time $\bar{q}$(t). The efficacy of condoms is given as *ϖ*.

The probability of transmission per partnership $Z_{l,k,p,s^{'},l^{'},k^{'},p^{'}}$ depends on (i) the probability of transmission per sex act, and (ii) the number of sex acts during the partnership (which depends on the risk group of each partner). The probability of transmission per sex act depends on an individual’s circumcision status (if male), in addition to their partner’s state of HIV infection (including ART use), circumcision status (if male) and the degree of condom use in the partnership (which depends on the risk group of each partner).

A baseline transmission probability from uncircumcised males to females is assumed (β_0_). The difference in acquisition and transmission per sex act for other factors (e.g. stage of infection) is specified with respect to this baseline transmission probability using a multiplicative factor. The probability of HIV transmission per sex act is given by $\beta_{k}^{s^{'}k^{'}}$and depends on: s’ (partner’s HIV status), k’ (partner’s circumcision status (if male)), and k (individual’s circumcision status (if male)). The probability of transmission from males to females is assumed to be identical to that for transmission from females to males. Male circumcision is assumed to reduce the risk of acquisition but not onward transmission.

##### Sexual mixing

The mixing pattern is defined with respect to sex, five-year age group and behavioural risk group. The proportion of sexual partnerships that an individual of sex g (where g=1 refers to females and g=2 to males), 5 year age group A and risk group *l* forms with an individual of the opposite sex, age group A*’* and risk group *l’*, is given by $\rho_{g,A,l,A^{'}l^{'}}$, and is defined as:

Females:

$P_{1,A,l,A^{'}l^{'}}=\varepsilon_{A}\varepsilon_{l}\left( \delta_{A,A^{'}}\delta_{l,l^{'}} \right)$ assortative age and assortative risk

$+\left( 1-\varepsilon_{A} \right)\varepsilon_{l}\left( \delta_{l,l^{'}}\frac{C_{2,A^{'},l^{'}}\sum_{k^{'}=2}^{3} \sum_{p^{'}} \sum_{s^{'}} X_{l^{'},k^{'},A^{'}}^{s^{'},p^{'}}}{\sum_{A^{'}} C_{2,A^{'},l^{'}}\sum_{k^{'}=2}^{3} \sum_{p^{'}} \sum_{s^{'}} X_{l^{'},k^{'},A^{'}}^{s^{'},p^{'}}} \right)$ disassortative age, assortative risk

$+\varepsilon_{A}\left( 1-\varepsilon_{l} \right)\left( \delta_{A,A^{'}}\frac{C_{2,A^{'},l^{'}}N_{g^{'}}\left( A^{'},l^{'} \right)}{\sum_{l^{'}} C_{2,A^{'},l^{'}}\sum_{k^{'}=2}^{3} \sum_{p^{'}} \sum_{s^{'}} X_{l^{'},k^{'},A^{'}}^{s^{'},p^{'}}} \right)$ assortative age, disassortative risk

$+\left( 1-\varepsilon_{A} \right)\left( 1-\varepsilon_{l} \right)\left( \frac{C_{2,A^{'},l^{'}}N_{g^{'}}\left( A^{'},l^{'} \right)}{\sum_{A^{'}} \sum_{l^{'}} C_{2,A^{'},l^{'}}\sum_{k^{'}=2}^{3} \sum_{p^{'}} \sum_{s^{'}} X_{l^{'},k^{'},A^{'}}^{s^{'},p^{'}}} \right)$ disassortative age and risk

Males:

$P_{2,A,l,A^{'}l^{'}}=\varepsilon_{A}\varepsilon_{l}\left( \delta_{A,A^{'}}\delta_{l,l^{'}} \right)$ assortative age and risk

$+\left( 1-\varepsilon_{A} \right)\varepsilon_{l}\left( \delta_{l,l^{'}}\frac{C_{1,A^{'},l^{'}}\sum_{p^{'}} \sum_{s^{'}} X_{l^{'},1,A^{'}}^{s^{'},p^{'}}}{\sum_{A^{'}} C_{1,A^{'},l^{'}}\sum_{p^{'}} \sum_{s^{'}} X_{l^{'},1,A^{'}}^{s^{'},p^{'}}} \right)$ disassortative age, assortative risk

$+\varepsilon_{A}\left( 1-\varepsilon_{l} \right)\left( \delta_{A,A^{'}}\frac{C_{1,A^{'},l^{'}}\sum_{p^{'}} \sum_{s^{'}} X_{l^{'},1,A^{'}}^{s^{'},p^{'}}}{\sum_{l^{'}} C_{1,A^{'},l^{'}}\sum_{p^{'}} \sum_{s^{'}} X_{l^{'},1,A^{'}}^{s^{'},p^{'}}} \right)$ assortative age, disassortative risk

$+\left( 1-\varepsilon_{A} \right)\left( 1-\varepsilon_{l} \right)\left( \frac{C_{1,A^{'},l^{'}}\sum_{p^{'}} \sum_{s^{'}} X_{l^{'},1,A^{'}}^{s^{'},p^{'}}}{\sum_{A^{'}} \sum_{l^{'}} C_{1,A^{'},l^{'}}\sum_{p^{'}} \sum_{s^{'}} X_{l^{'},1,A^{'}}^{s^{'},p^{'}}} \right)$ disassortative age and risk

Note: $\sum_{A^{'}} \sum_{l^{'}} \rho_{g,A,l,A^{'},l^{'}}=1$

(10)

The parameter *C_g,A,l_* gives the mean number of partners in a year per individual of sex *g* in age group A and risk group *l*. The degree of assortativity in mixing with respect to age and with respect to risk group are given by ε_A_ and ε_l_, respectively. The identity matrix with respect to risk is given by *δ_l,l’_* whereby:

$$\delta_{l,l^{'}}=\left\{ \begin{aligned} 1, if l=l^{'} \\ 0, if l\neq l^{'} \end{aligned} \right.$$

(11)

A discrepancy matrix $D_{A_{2},l_{2},A_{1},l_{1}}$is defined to balance the number of sexual partnerships between males and females formed with respect to each age group and risk group, where *A_2_* and *l_2_* are the age and risk group of the male partner and *A_1_* and *l_1_* are the age and risk group of the female partner. It is calculated as follows:

$$D_{A_{2},l_{2},A_{1},l_{1}}= \frac{\rho_{2,A,l,A^{'}l^{'}}C_{2,A,l}\sum_{k=2}^{3} \sum_{p} \sum_{s} X_{l,k,A}^{s,p}}{\rho_{1,A,l,A^{'}l^{'}}C_{1,A,l}\sum_{p} \sum_{s} X_{l,1,A}^{s,p}}$$

(12)

The extent to which balancing of the number of sexual partnerships is male-driven is determined by parameter *θ*. When *θ=0.5* the sexes compromise equally. Balancing the number of sexual partnerships is carried out with respect to both partners’ age and risk groups and is represented by:

$$\rho_{2,A,l,A^{'}l^{'}}\longrightarrow D_{A_{2},l_{2},A_{1},l_{1}}{}^{(\theta-1)}{\rho_{2,A,l,A^{'}l^{'}}}$$

$$\rho_{1,A,l,A^{'}l^{'}}\longrightarrow D_{A_{2},l_{2},A_{1},l_{1}}{}^{(\theta)}{\rho_{1,A,l,A^{'}l^{'}}}$$

(13)

| **Parameter** | **Symbol** | **Value** | **Notes** |
| --- | --- | --- | --- |
| Fraction of women in “low” risk group | *ψf(1)* | Varied | Sampled for each pair of model runs |
| Fraction of women in “medium” risk group | *ψf(2)* | Varied | Sampled for each pair of model runs  Note all remaining women are assumed to be “high risk” |
| Fraction of men in low risk group | *ψm(1)* | Varied | Sampled for each pair of model runs |
| Fraction of men in medium risk group | *ψm(2)* | Varied | Sampled for each pair of model runs  Note all remaining men are assumed to be “high risk” |
| Balancing sexual partnerships | *θ* | 0.5 | Calibrated |
| Age assortativity in sexual partnership formation | ε_A_ | 0.8 | Calibrated |
| Risk group assortativity in sexual partnership formation | ε_l_ | 0.8272 | Calibrated |

Table S2: Behavioural parameters and values

The number of sex acts per partnership depends on behavioural risk group. The “low” risk groups are intended to reflect long-term stable partnerships and these are assumed to have a high number of sex acts overall. A value of 100 sex acts each year is assumed based on reported frequency of sex in marital relationships in Southern Africa [12].Those in the higher risk groups tend to form more partnerships, but each of these partnerships comprises fewer sex acts and higher condom use. A value of two sex acts is assumed as a representative assumption of casual and commercial sex.

**β_0_ adjustment**

We ran the model using different values of the true HR. In the absence of a correction factor, a higher HR would result in higher HIV prevalence, and therefore higher DALY burden. We required the baseline calibration to be independent of the true HR and therefore used a correction factor to reduce β_0,_ the baseline transmission probability such that the same epidemic fit could be recreated using different values of the true HR. Equation (14) shows the adjustment factor used to calculate β_0_. The constants *k_1_*, *k_2_*, *h_1_* and *h_2_* were calibrated such that the total DALYs accrued are approximately constant between baseline runs, regardless of the true HR.

$$\beta_{0}=\frac{\beta_{0 init}}{1+k_{1}(H-h_{1})}+|k_{2}\left( H-h_{2} \right)|$$

(14)

| **Parameter** | **Symbol** | **Value** | **Source** |
| --- | --- | --- | --- |
| Baseline transmission probability from uncircumcised males in the asymptomatic stage of HIV infection to females in a single act of unprotected sex | β_0_ |  | Calibrated for each model run. β_0_ decreases for increasing HR such that the DALYs generated by each baseline model runs are approximately equal, regardless of the sampled HR.  The parameter is representative and captures impact of other risk factors not explicitly models such as infection with STIs other than HIV [13,14]. |
| Constant | *k_1_* | 0.072 | Calibrated |
| Constant | *k_2_* | 3.41x10^-6^ | Calibrated |
| Constant | *h_1_* | 0.93 | Calibrated |
| Constant | *h_2_* | 1.175 | Calibrated |
| **Factor increase in transmission:** |  |  |  |
| To users of DMPA | $\beta_{3,1}^{s,k^{'}}$ | HR | Sampled from a distribution of hazard ratios. (See section 1.1) |
| From population with acute HIV infection | $\beta_{p,k}^{1,k^{'}}$ | 27 | [6] |
| From population with chronic HIV infection and CD4 >350 cells/μL | $\beta_{p,k}^{2,k^{'}}$ | 1 | The baseline transmission probability is assumed to apply from the end of acute infection until the period of heightened infectiousness 19-10 months before death [6]. |
| From population with chronic HIV infection and CD4 >200 cells/μL but <350 cells/μL | $\beta_{p,k}^{3,k^{'}}$ | 1.6 |  |
| From population in late infection | $\beta_{p,k}^{4,k^{'}}$ | 3.8 | [6] |
| From population in pre-AIDS | $\beta_{p,k}^{5,k^{'}}$ | 3.8 |  |
| From population in AIDS | $\beta_{p,k}^{6,k^{'}}$ | 3.8 |  |
| From population on early ART | $\beta_{p,k}^{8,k^{'}}$ | 0.08 | [7] |
| From population on late ART | $\beta_{p,k}^{10,k^{'}}$ | 0.08 | [7] |
| From population who have dropped out of ART | $\beta_{p,k}^{11,k^{'}}$ | 3.56 | Estimated |
| From women | $\beta_{p,k}^{s^{'},1}$ | 1 | Transmission from males to females is assumed to be the same as that from females to males [14]. |
| From uncircumcised men | $\beta_{p,k}^{s^{'},2}$ | 1 |  |
| From circumcised men | $\beta_{p,k}^{s^{'},3}$ | 1 | Assumes no effect of circumcision on  HIV transmission. |
| To circumcised men | $\beta_{p,3}^{s^{'},k^{1}}$ | 0.4 | Risk of HIV acquisition is 60% lower than among uncircumcised men [15–17] |
| Condom efficacy | ☐ | 0.1 | Assumes condoms provide 90% protection from HIV infection |

**Table S3: Factor increments in transmission probability per sex act with respect to baseline transmission probability (β_0_)**

# Male circumcision

The level of circumcision changes over time to reflect the increase in male circumcision as has occurred in recent years according to a nationally representative survey and is projected to continue increasing in the future [11] (Figure S2) . The movement of uncircumcised men to circumcised classes is represent by the function $g\left( k \right)$, included in the equations below.

$g\left( k \right)=\frac{dX_{l,k,a}^{0,p}}{dt}$ Change in circumcision status

$\frac{dX_{l,2,a}^{0,p}}{dt}=-\eta_{C}X_{l,2,a}^{0,p}$ Uncircumcised men

$\frac{dX_{l,3,a}^{0,p}}{dt}= \eta_{C}X_{l,2,a}^{0,p}$Circumcised men

(14)

The parameter $\eta_{C}$ gives the scale-up rate for male circumcision, which is a time-varying parameter based on the extent to which the current level of circumcision in the sexually active adult population matches the data on circumcision prevalence. Movement from uncircumcised to circumcised classes occurs at age 15 to represent circumcision that has occurred after birth but before entering the sexually active population. In the model, the rate of HIV acquisition for circumcised men is reduced by 60% [15–17].


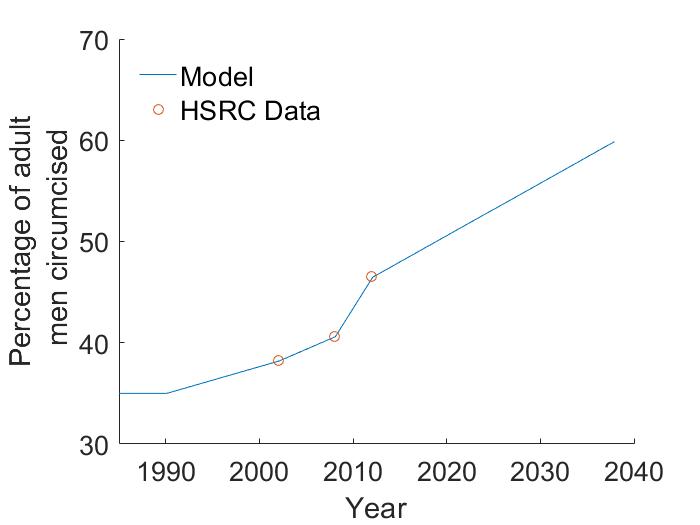


***Figure S2. The proportion of adult men that are circumcised with respect to time.***

*The level of circumcision in the model was calibrated to data reported in a nationally representative survey* [11]*.*

# Antiretroviral Treatment

ART can be initiated for the population with four programme types, specified with different initiation rules, as described above. A drop-out rate of 0.005 is assumed, regardless of the CD4 level at which ART is initiated.

A rate of progressing to AIDS of 0.0843 is assumed for those initiating ART below 200 cells/μL. A rate of progressing to AIDS of 0.0026 is assumed for those initiating ART above 200 cells/μL. The number of individuals receiving ART is calibrated to the total number of people on ART in South Africa [18,19] (Figure S3).


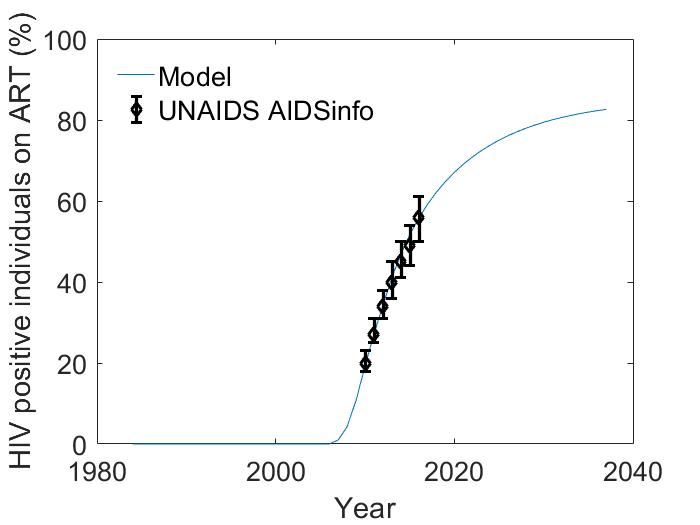


***Figure S3. The percentage of HIV positive adults (15-49) receiving antiretroviral therapy in South Africa.***

*Model data is compared to estimates of the percentage of HIV positive adults on ART in South Africa* [18,19]

# Contraception

We model the use of different contraceptives by splitting the female population into seven contraceptive compartments: no method, oral hormonal contraceptives, DMPA, norethisterone enanthate (NET-EN), copper intrauterine device (IUD), female sterilisation and ‘other’ methods.

| **Contraceptive** | **Efficacy (typical use)** | **One year discontinuation rates (%)** | **Source** |
| --- | --- | --- | --- |
| No method | 15% |  | [20] |
| Combined oral contraceptive | 91% | 33 | [20] |
| DMPA | 94% | 44 | [20] |
| NET-EN | 94% | 44 | Assumed to be the same as DMPA |
| Copper IUD | 99.2% | 22 | [20] |
| Female sterilisation | 99.5% |  | [20] |
| Other methods | 83.9% | 33 (assumed to be the same as oral contraceptives) | average of the efficacies of: male sterilisation, withdrawal, fertility awareness and male condoms [20] |

***Table S4. Contraceptive efficacy and continuation rates for methods used in the model.***

Condom use is modelled separately to female-controlled contraceptives; it is used to impact upon the rate of HIV transmission (section 2.4).

The equations describing movement between contraceptive classes are given below.

$$q\left( p \right)= \frac{{dX}_{l,k,a}^{s,p}}{dt} Change in contraceptive use$$

For *p* = 2:7

$$\frac{{dX}_{l,k,a}^{s,p}}{dt}= {\eta_{p}X}_{l,k,a}^{s,1}- \sigma_{p}X_{l,k,a}^{s,p}$$

For *p* = 1 (no method)

$$\frac{{dX}_{l,k,a}^{s,1}}{dt}= \sum_{p=2}^{7} \sigma_{p}X_{l,k,a}^{s,p}- \sum_{p=2}^{7} \eta_{p}X_{l,k,a}^{s,1}$$

(15)

Women move away from contraceptives to the “no method” compartment based on the discontinuation rates ( $\sigma_{p}$ , Table S4). The rate of uptake $\eta_{p}$ of each method is calibrated such that contraceptive prevalences approximate those reported in the South African National HIV Prevalence, Incidence and Behaviour Survey, 2012 [11].

*
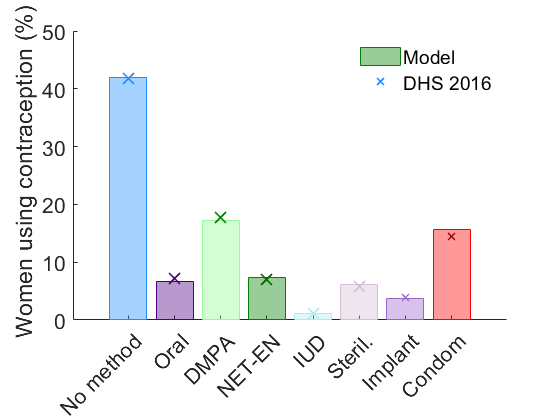
*

**Figure S4. Total contraceptive prevalence calibration**

*Total contraceptive prevalence among 15-49 year old women is calibrated to nationally representative survey data* [21]*.*

*
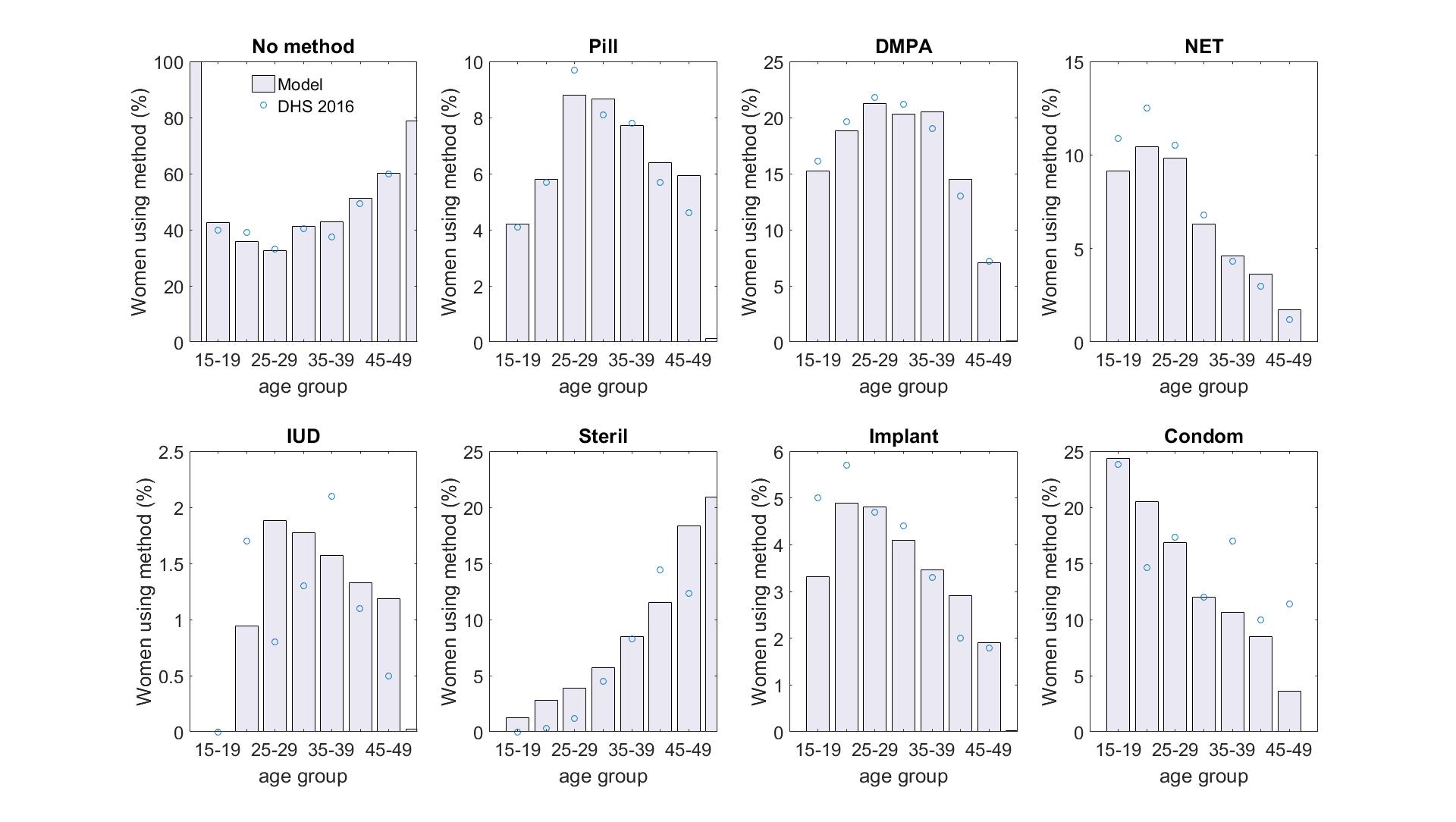
*

**Figure S5. Age-specific contraceptive prevalence calibration**

*Contraceptive prevalence among different age groups are calibrated to nationally representative survey data* [21]*.*

# Further Model calibration

The behavioural parameters and the baseline transmission probability were calibrated as these are difficult to empirically estimate reliably. Rates of contraceptive uptake were calibrated to fit contraceptive data. To account for the decrease in average fertility due to the addition of contraceptive classes, the baseline fertility rate was increased and calibrated using population size data. Calibrations are shown in supplementary Figures S2, S3, S4 and S5.


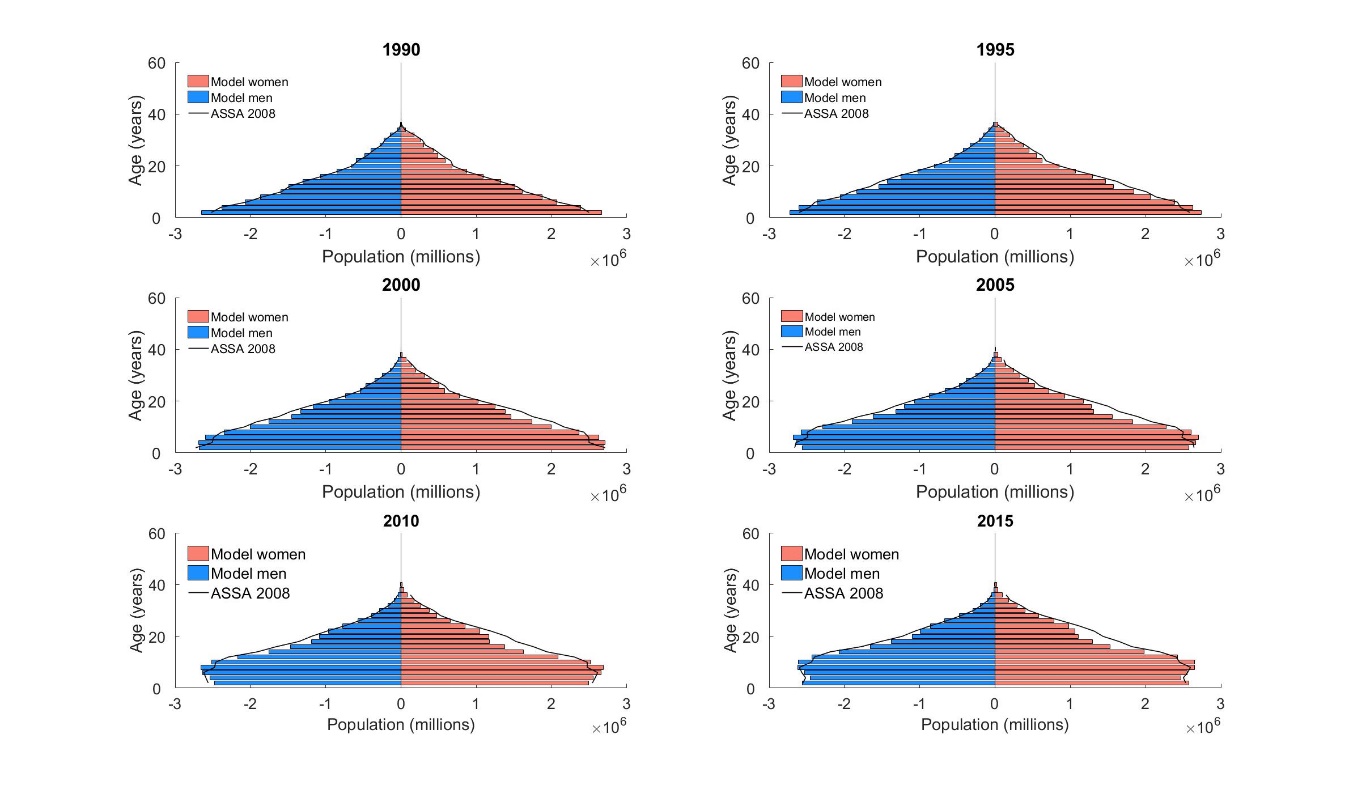


***Figure S6. Population pyramids for South Africa for 1985, 1990, 1995, 2000, 2005 and 2010.*** *Model population structure is compared to annual age-structured population size model estimates produced by the Actuarial Society of South Africa* [10]*.*

***
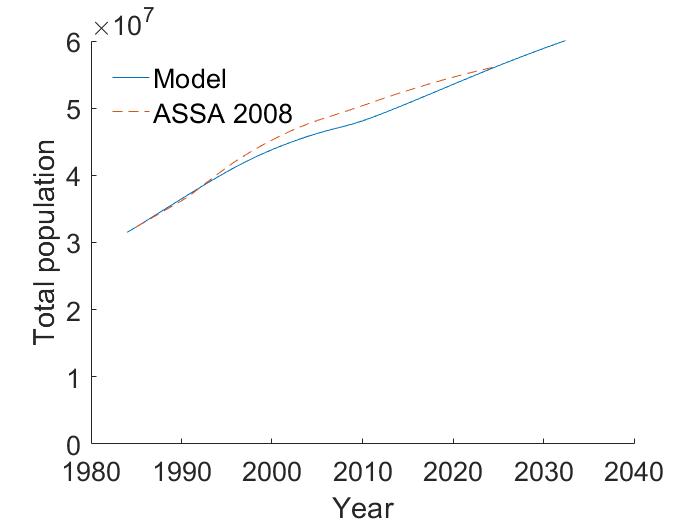
Figure S7. Population size with respect to time.***

*The total population of the model was calibrated to previous estimates from a demographic model of the South African population* [10]*.*


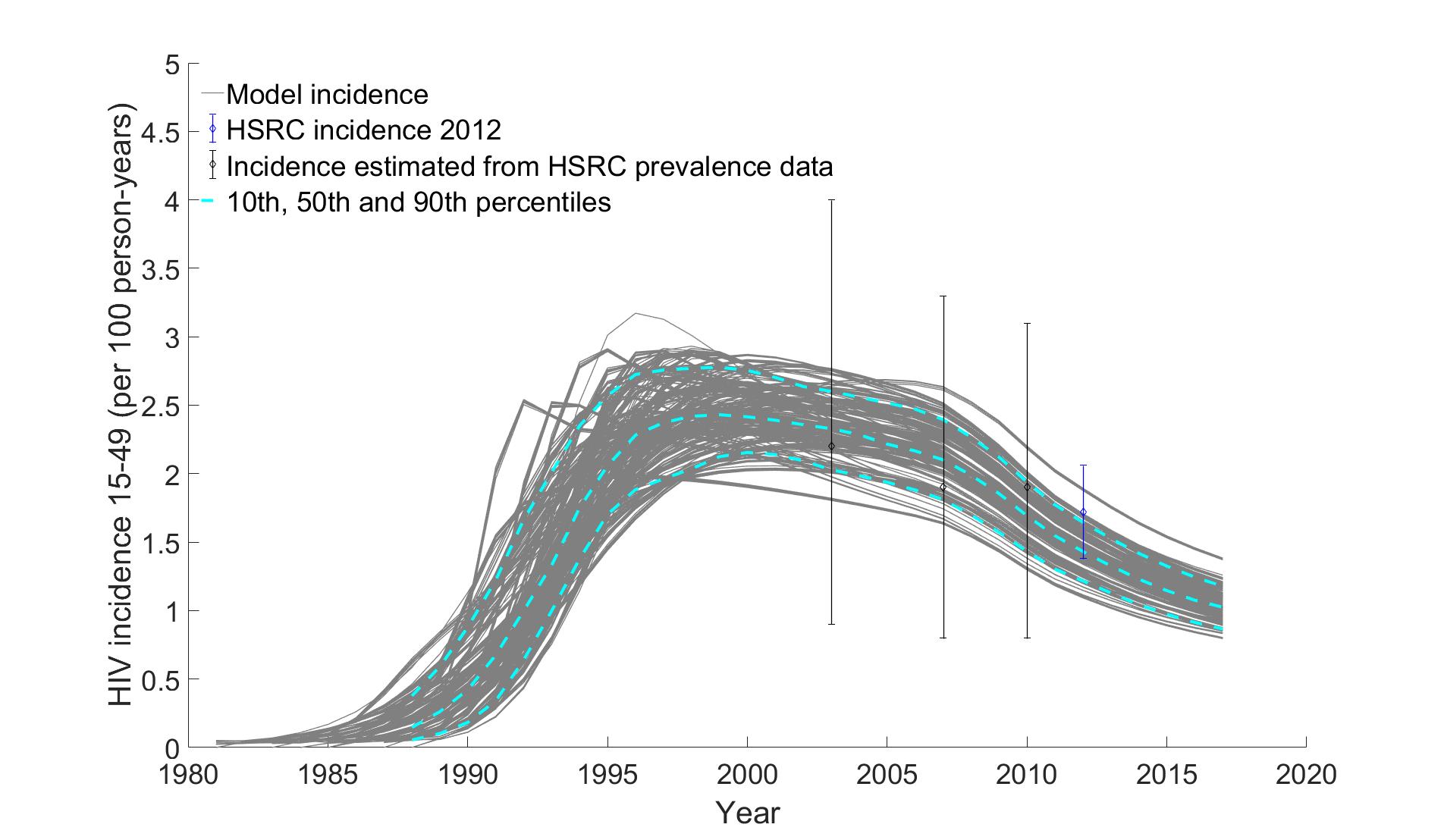


***Figure S8 HIV incidence in 15-49 year olds***

*HIV incidence in adults was calibrated to incidence data from a nationally representative survey as well as incidence estimates produced by a mathematical model calibrated to prevalence data* [11]*. Blue dotted lines represent 10^th^ , 50^th^ and 90^th^ percentiles of model variation.*

*
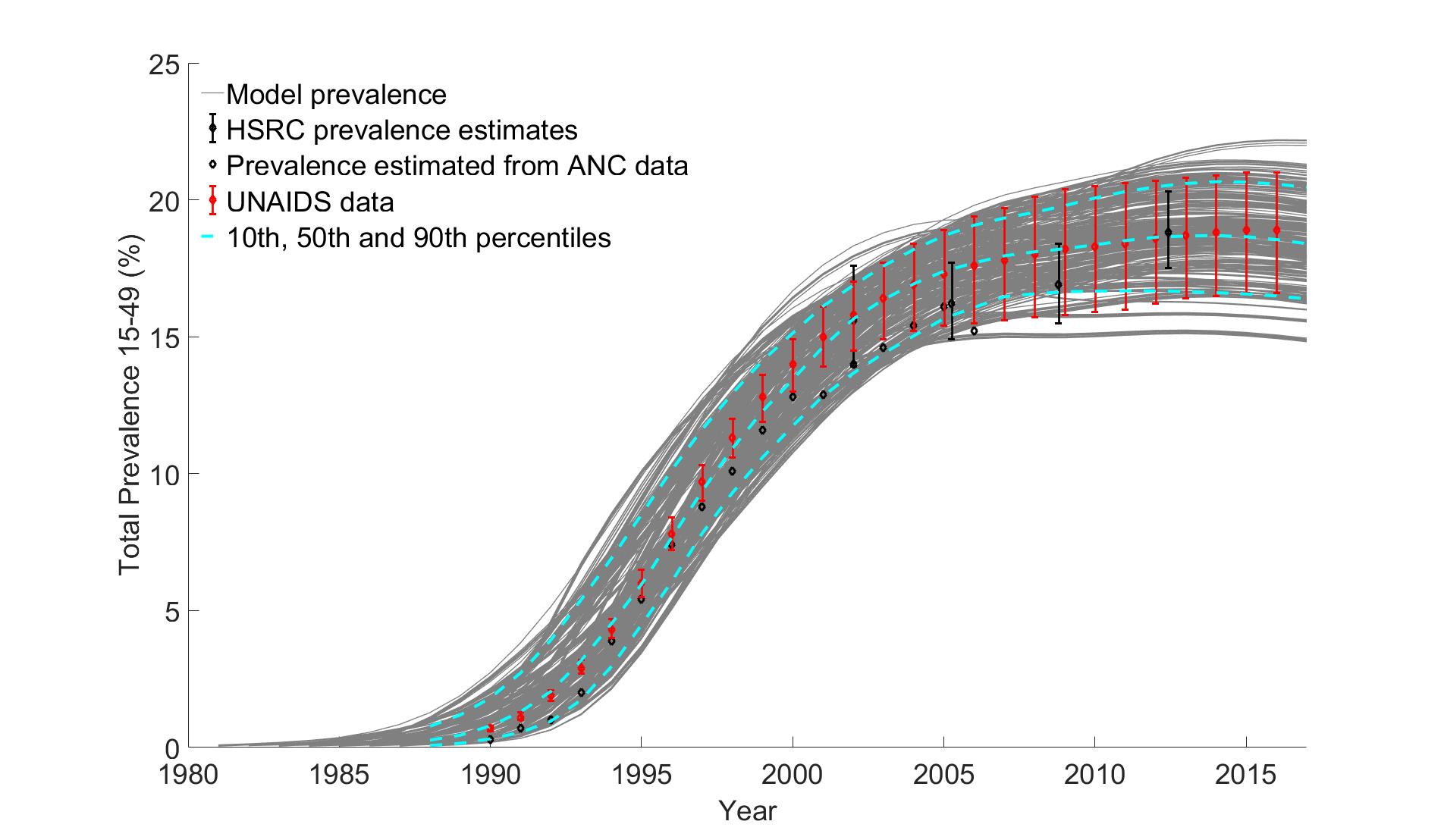
*

***Figure S9. Prevalence in 15-49 year olds.***

*Adult HIV prevalence is calibrated to nationally representative survey data from South Africa as well as UNAIDS prevalence estimates* [11,19,22]*. Blue dotted lines represent 10^th^ , 50^th^ and 90^th^ percentiles of model variation.*

*
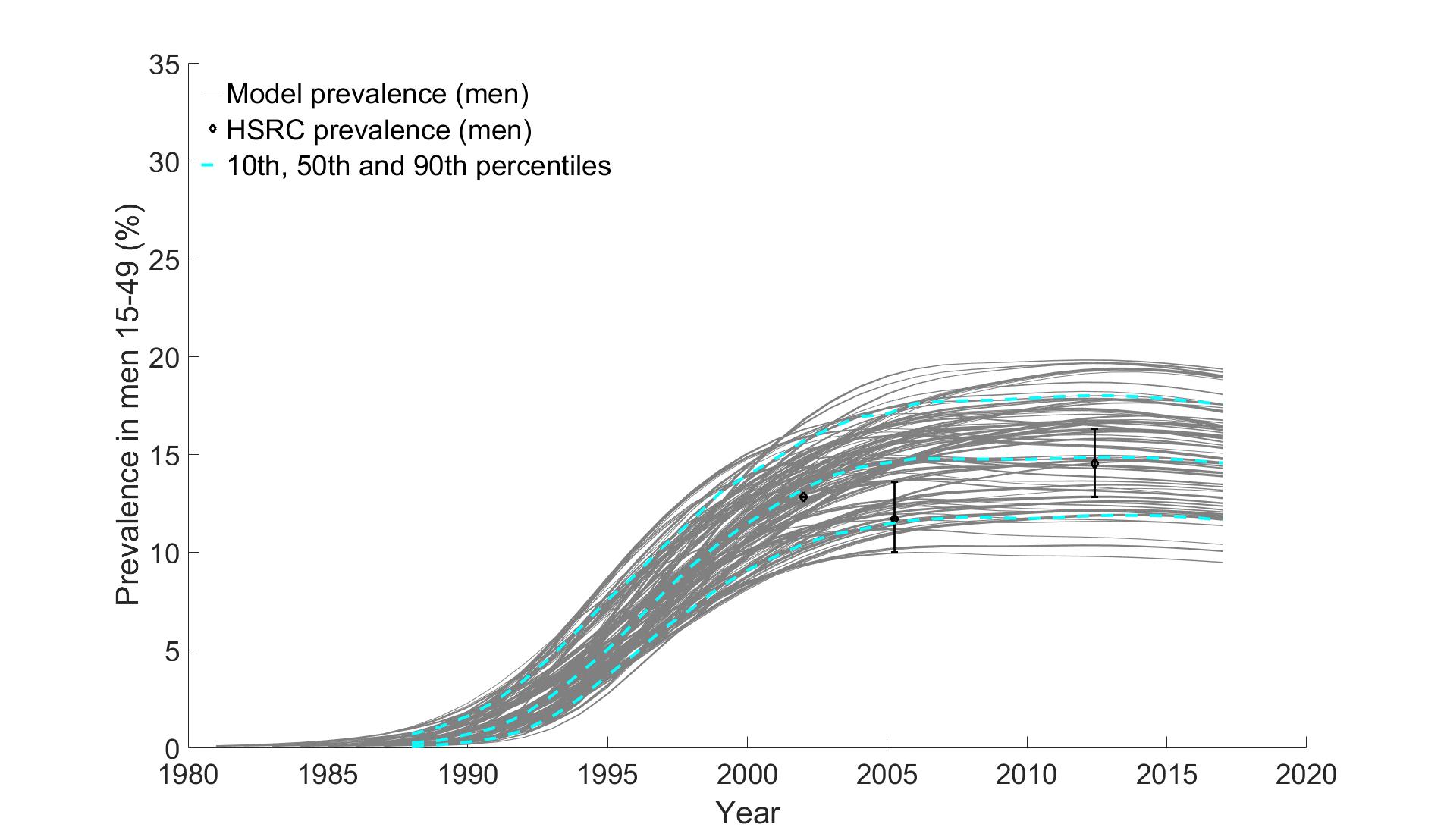
*

***Figure S10. Male HIV prevalence***

*HIV prevalence in the model was calibrated to sex-specific prevalence data* [11]*. Blue dotted lines represent 10^th^ , 50^th^ and 90^th^ percentiles of model variation.*

***
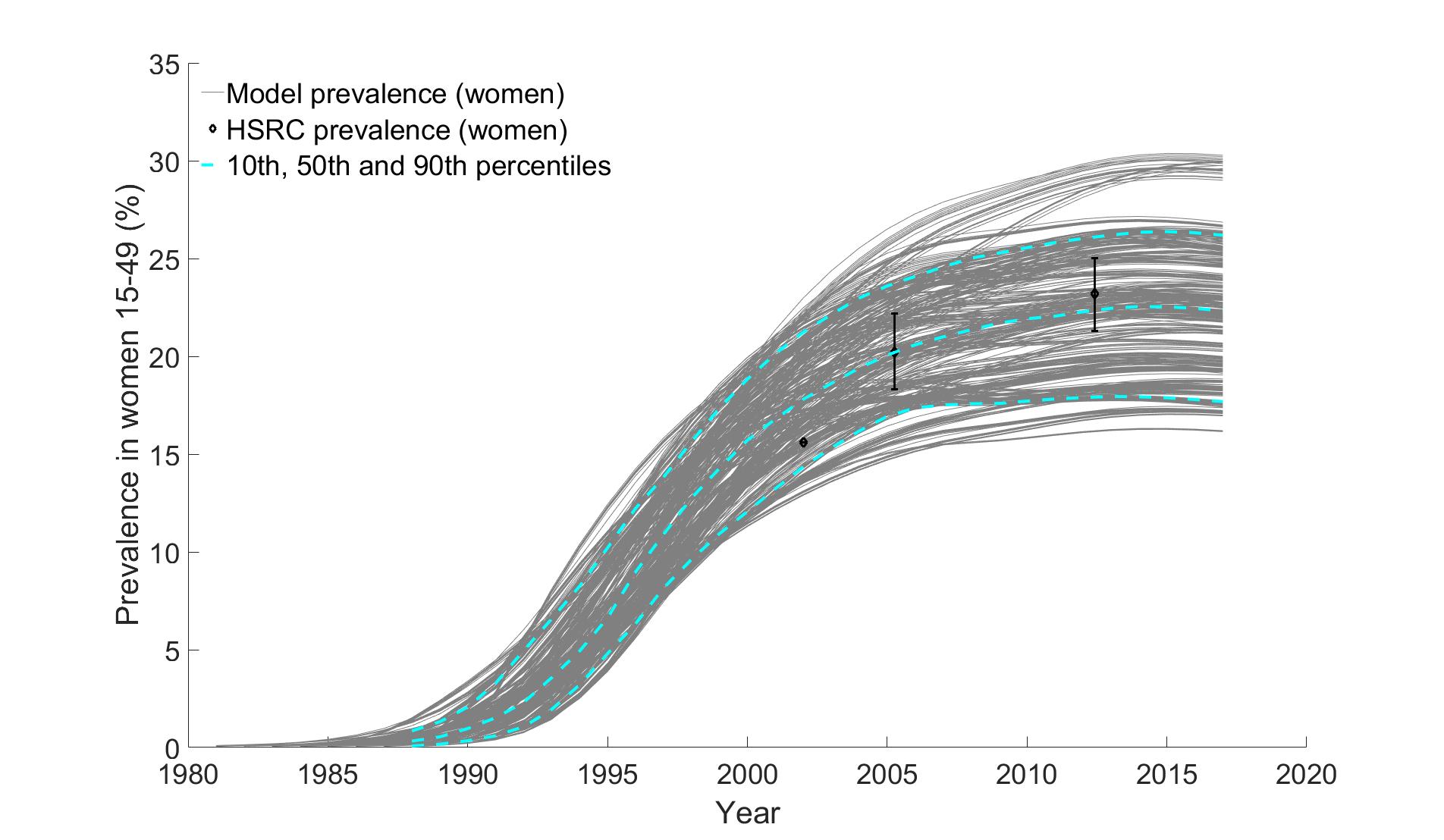
***

***Figure S11. Female HIV prevalence***

*HIV prevalence in the model was calibrated to sex-specific prevalence estimates* [11] *. Blue dotted lines represent 10^th^ , 50^th^ and 90^th^ percentiles of model variation.*

***
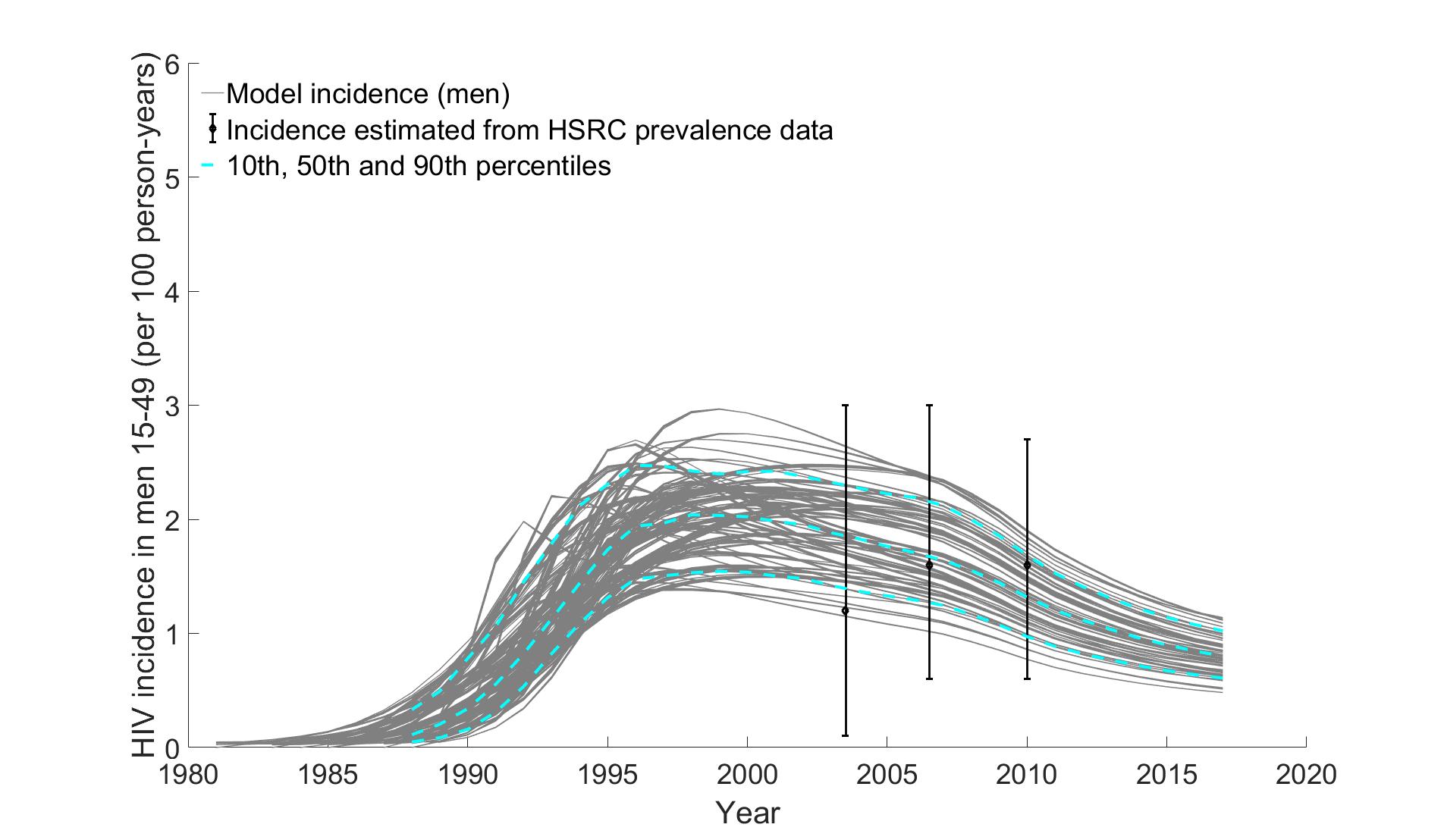
***

***Figure S12. Male HIV incidence***

*HIV incidence in the model was calibrated to sex-specific incidence estimates produced by a mathematical model calibrated to nationally representative prevalence data* [11]*. Blue dotted lines represent 10^th^ , 50^th^ and 90^th^ percentiles of model variation.*

***
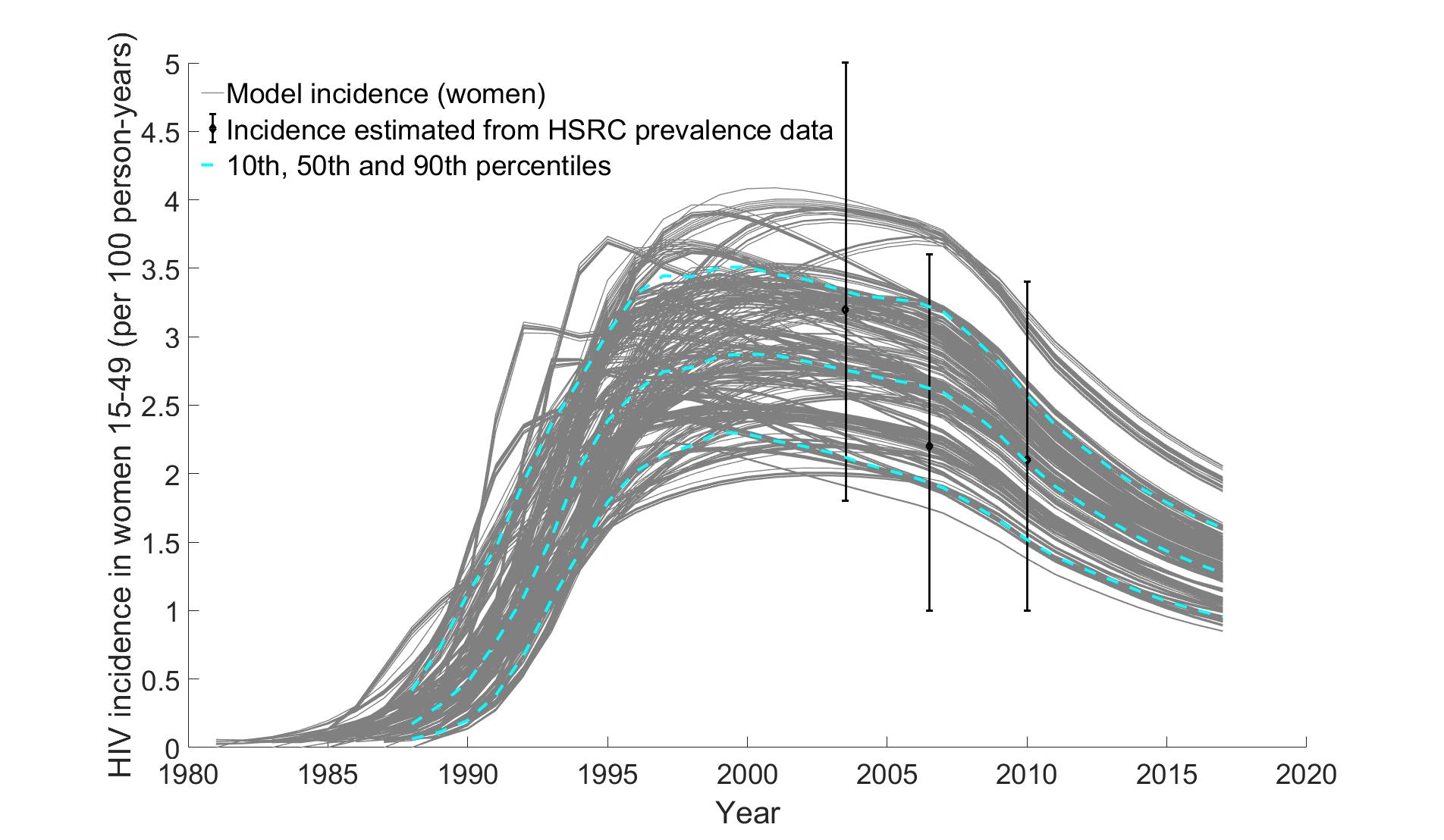
***

***Figure S13. Female HIV incidence***

*HIV incidence in the model was calibrated to sex-specific incidence estimates produced by a mathematical model calibrated to nationally representative prevalence data* [11]*. Blue dotted lines represent 10^th^ , 50^th^ and 90^th^ percentiles of model variation.*

# References

1 Cremin I, Alsallaq R, Dybul M, Piot P, Garnett G, Hallett TB. **The new role of antiretrovirals in combination HIV prevention: A mathematical modelling analysis**. *Aids* 2013; **27**:447–458.

2 Smith JA, Anderson SJ, Harris KL, McGillen JB, Lee E, Garnett GP, *et al.* **Maximising HIV prevention by balancing the opportunities of today with the promises of tomorrow: a modelling study**. *Lancet HIV* 2016; **3**:e289–e296.

3 Anderson RM, May RM. *Infectious diseases of humans: dynamics and control*. Oxford: Oxford University Press; 1991.

4 Garnett GP, Anderson RM. **Factors Controlling the Spread of HIV in Heterosexual Communities in Developing Countries: Patterns of Mixing between Different Age and Sexual Activity Classes**. *Philos Trans R Soc London B Biol Sci* 1993; **342**.

5 Garnett GP, Anderson RM. **Sexually transmitted diseases and sexual behavior: insights from mathematical models.** *J Infect Dis* 1996; :S150-61.

6 Hollingsworth TD, Anderson RM, Fraser C. **HIV-1 transmission, by stage of infection.** *J Infect Dis* 2008; **198**:687–93.

7 Donnell D, Baeten JM, Kiarie J, Thomas KK, Stevens W, Cohen CR, *et al.* **Heterosexual HIV-1 transmission after initiation of antiretroviral therapy: a prospective cohort analysis.** *Lancet* 2010; **375**:2092–8.

8 Cohen MS, Chen YQ, McCauley M, Gamble T, Hosseinipour MC, Kumarasamy N. **Prevention of HIV-1 Infection with Early Antiretroviral Therapy**. *N Engl J Med* 2011; **365**:493–505.

9 Lodi S, Phillips A, Touloumi G, Geskus R, Meyer L, Thiébaut R, *et al.* **Time from human immunodeficiency virus seroconversion to reaching CD4+ cell count thresholds <200, <350, and <500 Cells/mm3: Assessment of need following changes in treatment guidelines**. *Clin Infect Dis* 2011; **53**:817–825.

10 Actuarial Society of South Africa. **ASSA 2008 Model**. 2011.

11 Shisana O, Rhele T, Simbayi LC, Zuma K, Jooste S, Zungu N, *et al.* *South African National HIV Prevalence, Incidence and Behaviour Survey, 2012*. Cape Town: HSRC Press; 2012.

12 Brown MS. **Coitus, the proximate determinant of conception: inter-country variance in sub-Saharan Africa.** *J Biosoc Sci* 2000; **32**:145–159.

13 Boily M-C, Baggaley RF, Wang L, Masse B, White RG, Hayes RJ, *et al.* **Heterosexual risk of HIV-1 infection per sexual act: systematic review and meta-analysis of observational studies.** *Lancet Infect Dis* 2009; **9**:118–29.

14 Wawer MJ, Gray RH, Sewankambo NK, Serwadda D, Li X, Laeyendecker O, *et al.* **Rates of HIV-1 transmission per coital act, by stage of HIV-1 infection, in Rakai, Uganda.** *J Infect Dis* 2005; **191**:1403–9.

15 Bailey RC1, Moses S, Parker CB, Agot K, Maclean I, Krieger JN, Williams CF, Campbell RT N-AJ. **Male circumcision for HIV prevention in young men in Kisumu, Kenya: a randomised controlled trial.** *Lancet* 2007; **369**:643–656.

16 Auvert B, Taljaard D, Lagarde E, Sobngwi-Tambekou J, Sitta R, Puren A. **Randomized, controlled intervention trial of male circumcision for reduction of HIV infection risk: The ANRS 1265 trial**. *PLoS Med* 2005; **2**:1112–1122.

17 Gray RHM, Kigozi G, Serwadda D, Makumbi F, Watya S, Nalugoda F, *et al.* **Male circumcision for HIV prevention in men in Rakai, Uganda: a randomised trial**. *Lancet* 2007; **369**:657–666.

18 Johnson, Francis L. **Access to antiretroviral treatment in South Africa, 2004 - 2011**. *South Afr J HIV Med* 2012; **13**:22–27.

19 UNAIDS. UNAIDS AIDSinfo [Internet]. 2019 [cited 2019 Jan 21]. Available from: http://aidsinfo.unaids.org/

20 Trussell J. **Contraceptive Efficacy**. In: *Contraceptive Technology*.New York: Ardent Media; 2011. pp. 779–863.

21 National Department of Health (NDoH), Statistics South Africa (Stats SA) SAMR, Council (SAMRC) and I. *South Africa Demographic and Health Survey 2016: Key Indicators*. Pretoria, South Africa, and Rockville, Maryland, USA: NDoH, Stats SA, SAMRC, and ICF; 2017.

22 Granich RM, Gilks CF, Dye C, Cock KM De, Williams BG. **Universal voluntary HIV testing with immediate antiretroviral therapy as a strategy for elimination of HIV transmission : a mathematical model**. *Lancet* 2009; **373**:48–57.
